# NFAT-dependent and -independent exhaustion circuits program maternal CD8 T cell hypofunction in pregnancy

**DOI:** 10.1084/jem.20201599

**Published:** 2021-12-09

**Authors:** Emma L. Lewis, Rong Xu, Jean-Christophe Beltra, Shin Foong Ngiow, Jordana Cohen, Rahul Telange, Alexander Crane, Deirdre Sawinski, E. John Wherry, Paige M. Porrett

**Affiliations:** 1 Department of Obstetrics and Gynecology, The University of Pennsylvania, Philadelphia, PA; 2 Department of Surgery, The University of Pennsylvania, Philadelphia, PA; 3 Department of Systems Pharmacology and Translational Therapeutics, The University of Pennsylvania, Philadelphia, PA; 4 Institute for Immunology, University of Pennsylvania, Philadelphia, PA; 5 Parker Institute for Cancer Immunotherapy, University of Pennsylvania, Philadelphia, PA; 6 Department of Medicine, The University of Pennsylvania, Philadelphia, PA; 7 Department of Surgery, The University of Alabama at Birmingham, Birmingham, AL; 8 Comprehensive Transplant Institute, The University of Alabama at Birmingham, Birmingham, AL

## Abstract

Pregnancy is a common immunization event, but the molecular mechanisms and immunological consequences provoked by pregnancy remain largely unknown. We used mouse models and human transplant registry data to reveal that pregnancy induced exhausted CD8 T cells (Preg-T_EX_), which associated with prolonged allograft survival. Maternal CD8 T cells shared features of exhaustion with CD8 T cells from cancer and chronic infection, including transcriptional down-regulation of ribosomal proteins and up-regulation of TOX and inhibitory receptors. Similar to other models of T cell exhaustion, NFAT-dependent elements of the exhaustion program were induced by fetal antigen in pregnancy, whereas NFAT-independent elements did not require fetal antigen. Despite using conserved molecular circuitry, Preg-T_EX_ cells differed from T_EX_ cells in chronic viral infection with respect to magnitude and dependency of T cell hypofunction on NFAT-independent signals. Altogether, these data reveal the molecular mechanisms and clinical consequences of maternal CD8 T cell hypofunction and identify pregnancy as a previously unappreciated context in which T cell exhaustion may occur.

## Teaser

Fetal alloantigens prime maternal CD8 T cells during pregnancy, but the fate and function of these cells after pregnancy remain unclear. These studies demonstrate that pregnancy-primed CD8 T cells differentiate into exhausted T cells (T_EX_) that share molecular circuitry with T_EX_ that arise in chronic infection and cancer.

## Introduction

CD8 T cells provide protective immunity from pathogens, destroy tumor cells, mediate autoimmunity, and reject organ transplants (Wong and Pamer, 2003; van der Leun et al., 2020; Liblau et al., 2002; Rocha et al., 2003). The mechanisms that govern CD8 T cell fate and differentiation are therefore relevant to a variety of clinical disease areas. Of particular interest is how CD8 T cell function is tuned during differentiation, as CD8 T cells can develop into either canonical memory CD8 T cell populations with enhanced function (T_MEM_) or exhausted CD8 T cells (T_EX_) with diminished functional capacity (Lalvani et al., 1997; Veiga-Fernandes et al., 2000; Olson et al., 2013; Wherry et al., 2007; Thommen and Schumacher, 2018; McLane et al., 2019). Antigen dose and duration as well as costimulation and cytokine milieu are key signals that influence CD8 T cell fate (Iezzi et al., 1998; Shedlock and Shen, 2003; Kolumam et al., 2005; Mescher et al., 2006; Williams et al., 2006; Joshi et al., 2007; Ruby et al., 2007). However, as signals governing CD8 T cell differentiation may differ depending on the context of antigen encounter, it is unclear whether knowledge derived from models of infection or cancer can be generalized to CD8 T cell responses in other settings. This knowledge gap is particularly relevant for CD8 T cell differentiation in less well–studied areas such as the maternal T cell response to the fetus, despite the fact that exposure to fetal tissue antigens during pregnancy is the most common alloimmunization event in humans (Triulzi et al., 2009).

Pregnancy alloimmunization occurs when the maternal adaptive immune system is primed by paternally derived alloantigens that originate from the fetus and/or placenta. Priming may occur at either the maternal–fetal interface or within the maternal secondary lymphoid organs due to dissemination of alloantigens in the maternal circulation (i.e., fetal cells [Bianchi et al., 1996; Rijnink et al., 2015], cell-free DNA [Lo et al., 1997], and exosomes [Mitchell et al., 2015]). Whereas studies in mice (Erlebacher et al., 2007; Moldenhauer et al., 2009; Perchellet et al., 2013) and humans (van Kampen et al., 2001; James et al., 2003; Tilburgs et al., 2010; Lissauer et al., 2012) demonstrate that maternal T cells are activated by paternal alloantigens, the fate and function of these antigen-experienced T cells remain unclear. Although several studies suggest deletion and/or dysfunction of fetal-specific maternal CD8 T cells (Jiang and Vacchio, 1998; Erlebacher et al., 2007, Perchellet et al., 2013; Barton et al., 2017; Suah et al., 2021), other studies suggest that CD8 T cells can either maintain function (Norton et al., 2010) or have split function (Kinder et al., 2020), where some cells maintain function while others become less functional. In humans, decidual CD8 T cells in the uterus display a mixed transcriptomic signature of activation and dysfunction (Powell et al., 2017; van der Zwan et al., 2018), whereas CD8 T cells in the blood demonstrate cytotoxicity against both major (van Kampen et al., 2001) and minor (Lissauer et al., 2012) human alloantigens. However, few studies to date have addressed the molecular mechanisms governing maternal CD8 T cell fate or have compared antigen-experienced maternal T cells with other antigen-experienced populations. As a result, questions remain about the differentiation state of maternal fetal-specific CD8 T cells primed during pregnancy. Are fetal-specific CD8 T cells bona fide T_MEM_ cells capable of mediating rapid recall responses, or do they adopt a different fate? How does the differentiation state of these cells compare with other known CD8 T cell types? Because CD8 T cells primed by fetal alloantigens persist in the maternal repertoire and may participate in immune responses to pathogens, tumors, or transplant alloantigens later in life, answers to these questions could have implications for the clinical care of women during and after pregnancy.

In this work, we interrogated these questions by examining maternal CD8 T cell fate after pregnancy and the consequences of pregnancy alloimmunization. Using a mouse model in which fetal-specific maternal CD8 T cells could be identified, we found that fetal antigen drives the differentiation of hypofunctional CD8 T cells that persisted in the maternal repertoire. Persistence of these fetal-specific CD8 T cells primed during pregnancy correlated with prolonged graft survival in parous mice and humans. Moreover, these maternal CD8 T cells were hypofunctional and expressed multiple phenotypic and transcriptional features of T cell exhaustion, including PD-1 and TOX. Transcriptomic studies suggested that translational repression, a common feature of T_EX_ in chronic infection and cancer, was a major mechanism of pregnancy-induced T cell exhaustion. Pregnancy-induced fetal antigen–specific CD8 T cell exhaustion was driven by a previously described NFAT-dependent transcriptional circuit but also included a prominent imprint of NFAT-independent mechanisms. Altogether, our study identifies pregnancy as a previously unappreciated context in which CD8 T cell exhaustion occurs and highlights potential impacts of pregnancy on immunological responses in postpartum life.

## Results

### Pregnancy alloimmunization generates antigen-experienced CD8 T cells with impaired functionality

We sought to understand maternal CD8 T cell fate in a clinically relevant animal model where fetal-specific CD8 T cells could be identified and compared with T_MEM_ cells with identical antigen specificity. As polyclonal maternal T cell responses are driven by diverse tissue antigens derived from the polymorphic paternal histocompatibility complex (i.e., HLA in humans, MHC in mice), our investigations required the use of a surrogate fetal antigen that could prime maternal T cells of known specificity. The Act-mOVA/OT-1 transgenic system satisfied these criteria, as the SIINFEKL epitope of chicken OVA is bound to H-2K^b^ and expressed as a tissue antigen in Act-mOVA mice, and this antigen can effectively prime transgenic OT-1 CD8 T cells (Ehst et al., 2003). Pregnancies sired by Act-mOVA mice can prime adoptively transferred OT-1 TCR transgenic T cells in the maternal spleen (Erlebacher et al., 2007; Moldenhauer et al., 2009; Barton et al., 2017). Moreover, skin grafts from Act-mOVA donor mice prime OT-1 T cells to differentiate into cytokine-producing functional T_MEM_ cells (Bozeman et al., 2018). Thus, the use of an Act-mOVA model with adoptively transferred OT-1 cells provided a system to evaluate CD8 T cell priming during pregnancy and compare this priming with the development of T cell memory in a model of organ transplantation where tissue-derived antigens drive the CD8 T cell response.

**Figure 1.**
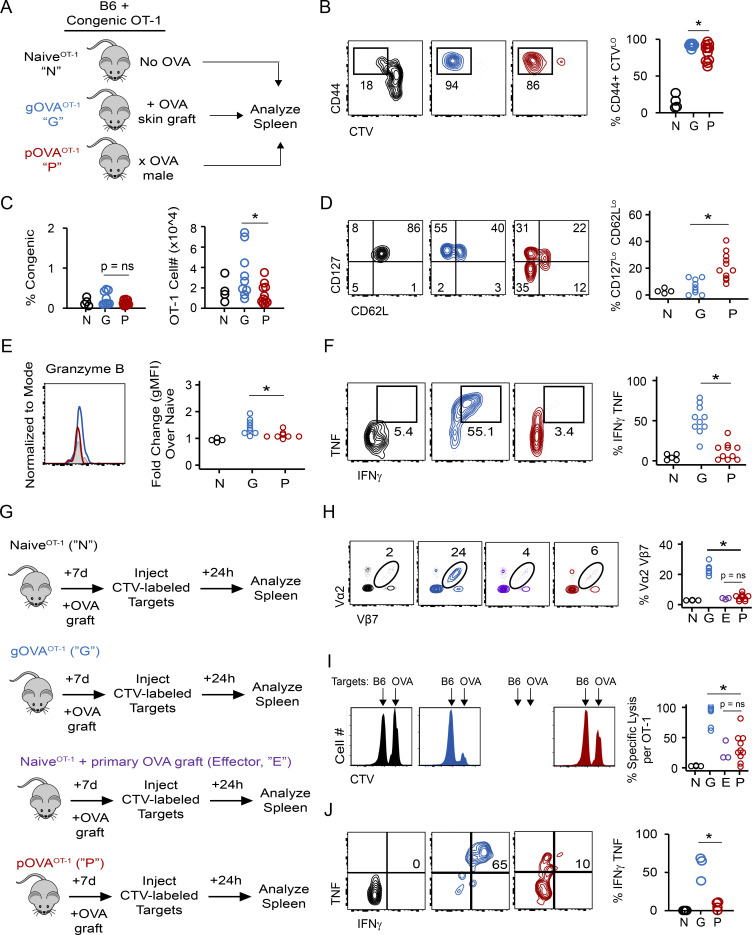
**Parous CD8 T cells are hypofunctional effector memory T cells.** **(A)** Experimental schematic for B–F. 3 × 10^6^ CTV-labeled CD45.2 OT-1 T cells were adoptively transferred into CD45.1 congenic mice. Mice received no OVA exposure (Naive^OT-1^ [N]), received an OVA skin graft (gOVA^OT-1^ [G]), or were mated with an Act-mOVA male (pOVA^OT-1^ [P]). Spleens were analyzed on days 50–60. Flow plots and histograms are gated on OT-1 CD8 T cells and derive from individual representative mice. With the exception of B and C, all other plots are additionally gated on CTV^Hi^, CD44^Lo^ cells (Naive^OT-1^ [black or gray]) or gated on CTV^Lo^, CD44^Hi^ cells (gOVA^OT-1^ [blue], pOVA^OT-1^ [red]). Data are pooled from two independent experiments. Circles represent individual mice; Naive^OT-1^, *n* = 4; gOVA^OT-1^, *n* = 9; pOVA^OT-1^, *n* = 11. Statistical comparisons were performed with Student’s *t* test. **(B)** OT-1 T cells are divided, antigen-experienced cells in OVA-exposed mice. *, P = 0.02. **(C)** Similar number and frequency of OT-1 T cells recovered from groups of mice. *, P < 0.04. **(D)** OT-1 T cells recovered from OVA-parous and OVA-grafted mice differ with respect to effector memory versus central memory phenotype. *, P < 0.001. **(E)** Granzyme B expression on OT-1 T cells. *, P = 0.01. gMFI, geometric mean fluorescence intensity. **(F)** Cytokine production from OT-1 cells. Splenocytes from naive^OT-1^, gOVA^OT-1^, and pOVA^OT-1^ mice were stimulated with OVA SIINFEKL peptide directly ex vivo. *, P < 0.001. **(G)** Experimental schematic for H–J. Naive^OT-1^, gOVA^OT-1^, and pOVA^OT-1^ mice were generated as in A, except OT-1 cells were not CTV labeled. 50–60 d later, gOVA^OT-1^ and pOVA^OT-1^ received an OVA skin graft. A group of naive^OT-1^ mice also received an OVA skin graft (Naive^OT-1^ + primary OVA graft [Effector]). 7 d after skin grafting, mice were injected with CTV-labeled target cells derived from either B6 syngeneic mice (CTV^lo^) or Act-mOVA mice (CTV^bright^). Spleens were analyzed 24 h later. Flow plots in H and J are gated on OT-1 CD8 T cells and derive from individual representative mice. Histograms derive from individual representative mice and are gated on CTV-labeled target cells. Data are pooled from two independent experiments. Circles represent individual mice. Naive^OT-1^, *n* = 4; gOVA^OT-1^, *n* = 6; pOVA^OT-1^, *n* = 10; Naive^OT-1 (primary effector)^, *n* = 3; except for J, where cytokine production was not measured in primary effector OT-1 T cells. Statistical comparisons were performed with Student’s *t* test. **(H)** Expansion of OT-1 T cells. *, P < 0.001. **(I)** Percentage of specific lysis of OVA target cells. *, P < 0.001. **(J)** Cytokine production in OT-1 T cells. *, P < 0.03.

We adoptively transferred OT-1 CD8 T cells into C57BL/6 congenic mice and exposed these mice to OVA antigen through pregnancy or skin transplantation (Fig. 1 A). 7 wk later, we compared the phenotype and function of antigen-experienced OT-1 cells recovered from the spleens of mice immunized by either pregnancy (pOVA^OT-1^) or a skin graft (gOVA^OT-1^). Antigen-experienced OT-1 CD8 T cells in both gOVA^OT-1^ and pOVA^OT-1^ mice had divided extensively, and the vast majority of donor OT-1 T cells up-regulated CD44 (>80%; Fig. 1 B). Antigen-experienced OT-1 CD8 T cells also persisted in the spleens of both gOVA^OT-1^ and pOVA^OT-1^ mice, although the overall number of OT-1 T cells was slightly reduced in pOVA^OT-1^ compared with gOVA^OT-1^ mice (Fig. 1 C). As ∼15% of the OT-1 cells in pOVA^OT-1^ mice and ∼5% of the OT-1 cells in gOVA^OT-1^ mice remained naive (Fig. 1 B), we subsequently analyzed only CD44^+^ divided (i.e., CellTrace Violet–low [CTV^Lo^]) cells and found that the OT-1 population in parous mice contained many CD62L^Lo^ and CD127^Lo^ cells (Fig. 1 D). OT-1 T cells in parous mice also had reduced expression of transcription factors such as TCF-1 and FoxO1 that repress effector T cell differentiation (TCF-1: 56 ± 14% of naive [pOVA] versus 86 ± 9% of naive [gOVA], P < 0.002; FoxO1: 61 ± 2% of naive [pOVA] versus 83 ± 2% of naive [gOVA], P < 0.01; Zhou et al., 2010; Rao et al., 2012). Despite reduced expression of CD62L and CD127 by OT-1 T cells in pOVA^OT-1^ mice, these cells expressed less granzyme B (Fig. 1 E) and had significantly less cytokine production (Fig. 1 F) than OT-1 T cells in gOVA^OT-1^ mice when stimulated ex vivo with SIINFEKL peptide. Collectively, these results suggested that pregnancy alloimmunization promoted distinct differences in the number, differentiation state, and function of antigen-experienced CD8 T cells.

As OT-1 T cells in parous mice had diminished cytokine production when stimulated ex vivo, we next tested the hypothesis that recall responses would be impaired in OT-1 T cells in parous mice. We therefore assessed expansion and function of OT-1 cells primed in gOVA^OT-1^ and pOVA^OT-1^ mice during a recall challenge (Fig. 1 G). 56 d after skin graft (gOVA^OT-1^) or pregnancy (pOVA^OT-1^), these mice were rechallenged with an OVA skin graft. For comparison, two groups of mice either received OT-1 cells followed by skin graft (i.e., a primary T cell response; naive^OT-1^) or just received OT-1 but no graft (negative control). Recall responses of OT-1 cells in gOVA^OT-1^ and pOVA^OT-1^ mice were then compared with primary effector responses by examining the frequency of OT-1 T cells and cytotoxic capacity in naive^OT-1^ mice (Fig. 1 G, purple). OT-1 T cells in pOVA^OT-1^ mice had limited secondary expansion capacity (Fig. 1 H), impaired cytolytic function (Fig. 1 I), and diminished cytokine production (Fig. 1 J) compared with OT-1 T cells in gOVA^OT-1^ mice. Notably, whereas the OT-1 cells in the gOVA^OT-1^ mice mounted a robust secondary response, the response of antigen-experienced OT-1 T cells in pOVA^OT-1^ mice was more similar to the primary response observed in the naive^OT-1^ mice. These results highlight that although maternal CD8 T cells can acquire effector function when primed during pregnancy, these cells do not acquire the enhanced functionality and recall capacity typical of antigen-experienced T_MEM_.

### Clinical implications of T cell hypofunction after pregnancy alloimmunization

**Figure 2.**
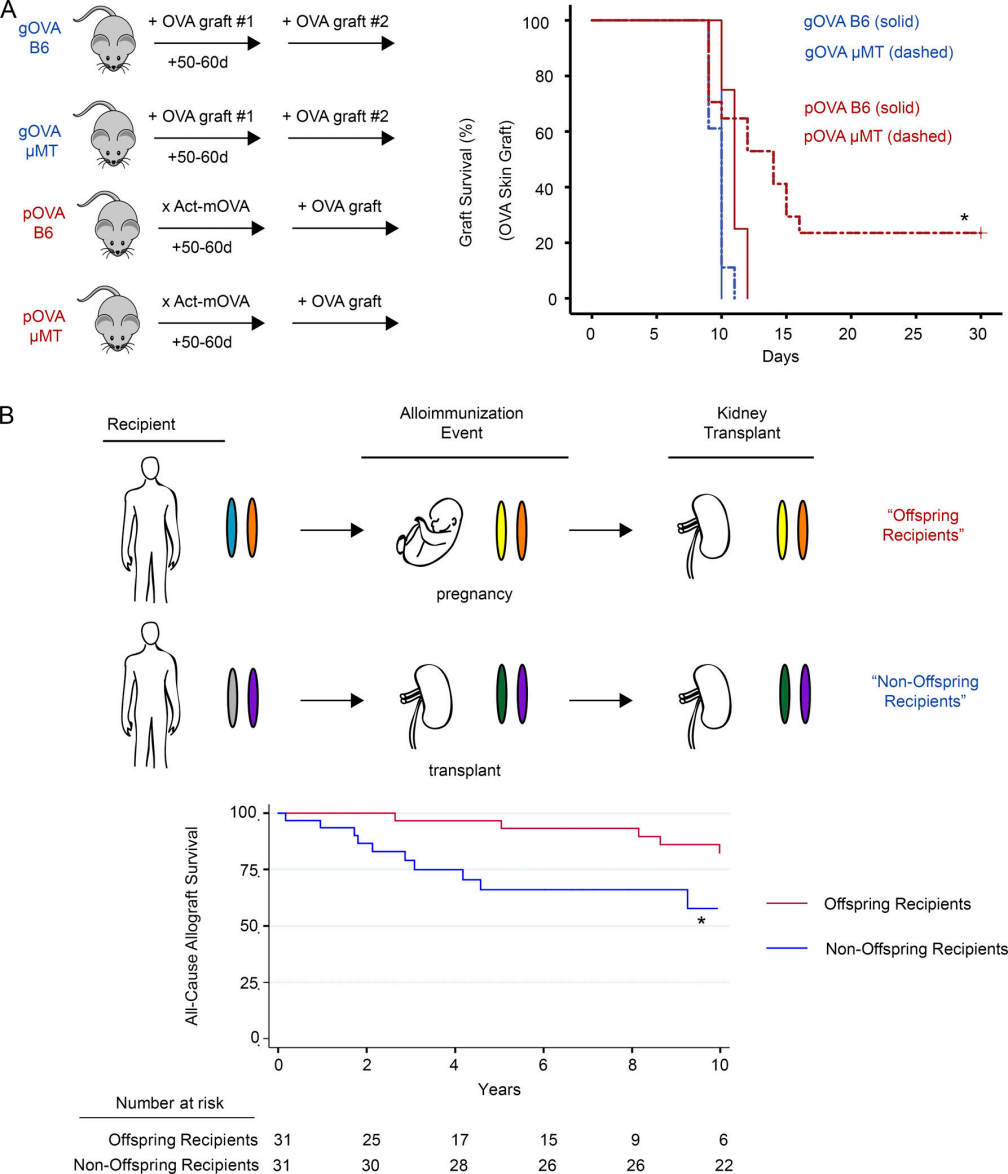
**Prolonged graft survival in recipients alloimmunized by prior pregnancy.** **(A)** Left: B6 and μMT mice were OVA alloimmunized through pregnancy (pOVA) or skin grafting (gOVA). Mice received an OVA skin graft 50–60 d subsequent to the initial immunization event. Right: OVA skin graft survival is prolonged in antibody-deficient mice alloimmunized by prior pregnancy. B6 gOVA, *n* = 4; μMT gOVA, *n* = 18; B6 pOVA, *n* = 4; μMT pOVA, *n* = 17. Pooled from two experiments. Kaplan-Meier survival analysis; log-rank test statistic. *, P = 0.001. **(B)** Top: Schematic of alloimmunization events and HLA haplotype of human kidney transplant recipients. Colored ovals depict paternal and maternal haplotypes. Offspring recipients (red) were alloimmunized by pregnancy and received a kidney transplant years later from the individual to whom they were originally alloimmunized. Offspring recipients were thus haplotype matched with both fetus and kidney donor (orange chromosome). Nonoffspring recipients (blue) were originally alloimmunized by a haplotype-matched kidney transplant and then received a second kidney transplant that was HLA identical to the first transplant. Note that similar to the offspring recipients, the nonoffspring recipients were haplotype matched with their kidney donors (purple chromosome). Bottom: Differences in graft survival between kidney transplant recipients alloimmunized by prior pregnancy or prior transplantation. Kaplan-Meier survival analysis; log-rank test statistic. *, P = 0.028.

The immunological consequences of pregnancy alloimmunization may be most apparent in transplant recipients, as these individuals can encounter alloantigens on the transplanted organ that were initially encountered during pregnancy (e.g., HLA proteins). Although allograft outcomes are worse for recipients who have been alloimmunized by a prior transplant and who possess T_MEM_ cells in the repertoire (Benichou et al., 2017), transplant outcomes in parous women are not as clear (Porrett, 2018). To test the hypothesis that maternal T cell hypofunction impacts graft survival after pregnancy alloimmunization, we compared OVA skin graft survival in pOVA versus gOVA mice. Consistent with our prior work (Barton et al., 2017), gOVA mice rejected OVA skin transplants faster than naive mice (mean survival time, 8 d [gOVA] versus 14 d [naive]; P < 0.01), and both pOVA and gOVA mice rejected OVA skin grafts with rapid kinetics (Fig. 2 A). These results suggested that either type of alloimmunization generated CD8 T cells capable of rejecting a skin graft. However, we considered the alternate possibility that anti-OVA antibody could also contribute to graft loss in this model. Although our prior investigations suggested that syngeneic mating of B6 female mice with Act-mOVA males generated relatively low amounts of anti-OVA antibody (Barton et al., 2017), another recent study of pregnancy alloimmunization in a heart transplant model reported a role for fetal-specific antibody (Suah et al., 2021). Moreover, either pregnancy or transplantation can result in humoral sensitization of human transplant recipients that may limit organ survival, though these effects may only be clinically significant in a minority of women (Hönger et al., 2013; Regan et al., 1991; Loupy and Lefaucheur, 2018; Van Rood et al., 1958; Porrett, 2018).

**Figure S1.**
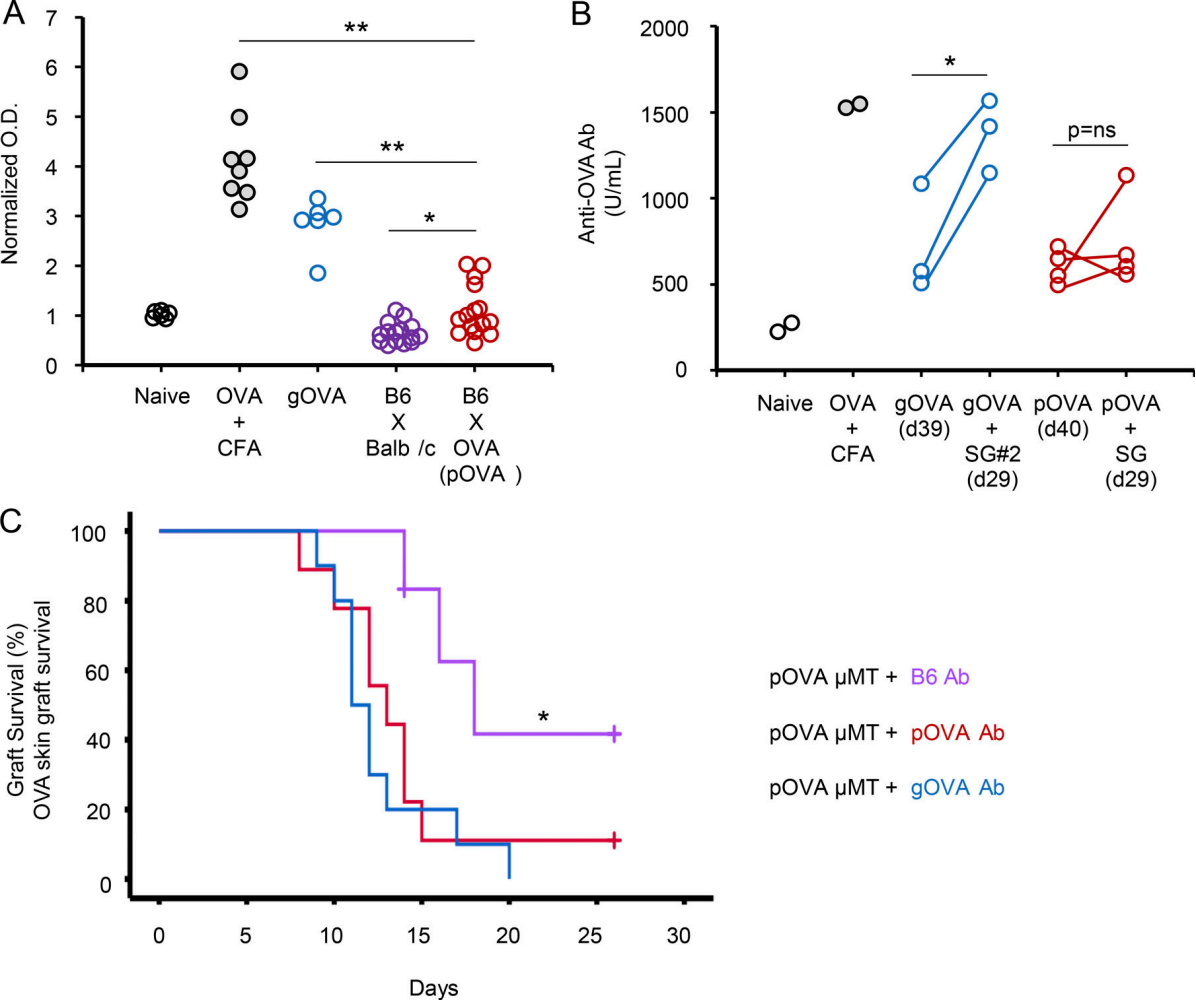
**Alloimmunization through pregnancy or transplantation results in graft-destructive antibody. (A)** Production of anti-OVA antibody after OVA immunization. Mice received (1) PBS injection with CFA (Naive; *n* = 6), (2) OVA peptide with CFA (gray circles; *n* = 8), or (3) an OVA skin graft (gOVA; blue circles; *n* = 6), or (4) were mated with allogeneic BALB/c males (B6xBALB/c; purple circles; *n* = 15), or (5) were mated with syngeneic Act-mOVA males (pOVA; red circles; *n* = 15). Anti-OVA antibody was measured in the sera with an ELISA ∼30 d after OVA immunization. Data were pooled from five experiments. *, P < 0.006; **, P < 0.001. **(B)** Anti-OVA antibody is increased after graft immunization. Sera were collected from gOVA and pOVA mice after initial OVA immunization. Mice then received an OVA skin graft, and sera were collected after grafting. Data are from one experiment with two to four mice per group. *, P < 0.03. **(C)** Restoration of accelerated skin graft rejection in pOVA μMT recipients after transfer of serum from pOVA and gOVA B6 mice. μMT female mice were mated with Act-mOVA males. 50–60 d after mating, mice received i.v. transfer of serum collected from pOVA (*n* = 9), gOVA (*n* = 10), or B6 naive (*n* = 6) mice. All mice then received an OVA skin graft. Serum was collected from B6 pOVA or B6 gOVA mice 50–60 d after initial OVA immunization. P = ns between pOVA μMT mice reconstituted via serum transfer from gOVA or pOVA B6 donors. Data are pooled from two experiments. *, P = 0.03.

Given this variability in humoral sensitization from pregnancy, we tested whether anti-OVA antibody was produced in pOVA or gOVA mice. In line with our prior work (Barton et al., 2017), the majority of pOVA mice made weak anti-OVA antibody (Fig. S1 A), with only 26% of pOVA mice developing more anti-OVA antibody than naive or BALB/c-mated parous mice (Fig. S1 A). Notably, the amount of antibody generated in pOVA mice was significantly lower than in mice immunized by OVA by other means (Fig. S1 A). To examine this issue in more detail, we quantified anti-OVA antibody in gOVA and pOVA mice before and after placement of an OVA skin graft using a quantitative antibody assay (Fig. S1 B). Anti-OVA antibody levels increased significantly in gOVA mice after receipt of a second graft, whereas antibody levels in pOVA mice remained relatively constant after OVA skin grafting (Fig. S1 B). Although anti-OVA antibody in pOVA mice was infrequent and was produced at a lower level than in gOVA mice, we directly tested the contribution of anti-OVA antibody to graft rejection by transferring serum from gOVA and pOVA mice into pOVA μ-membrane targeted deletion (μMT) mice (Fig. S1 C). Indeed, serum from either gOVA or pOVA donor mice accelerated graft rejection in pOVA μMT mice, pointing to a role for antibody in the pOVA setting. Altogether, these experiments suggested that anti-OVA antibody can promote and/or augment skin graft loss in pOVA mice, and they support the notion that relevant differences in OVA-specific CD8 T cells in pOVA and gOVA priming settings might still exist.

Despite this role of antibody, CD8 T cells can also mediate graft loss (Rocha et al., 2003). However, because pregnancy may induce hypofunctional antigen-experienced CD8 T cells (Fig. 1), we hypothesized that graft survival would be prolonged in parous animals when antibody was absent and graft rejection was exclusively T cell dependent. We therefore grafted OVA skin transplants onto gOVA μMT and pOVA μMT mice that lacked B cells and therefore could not make antibodies, and we monitored graft survival (Fig. 2 A). OVA skin grafts were rejected with equivalent tempo in either antibody-sufficient B6 gOVA mice or antibody-deficient gOVA µMT mice (Fig. 2 A). In contrast, we observed prolonged graft survival in 38% of antibody-deficient pOVA mice (pOVA µMT) compared with antibody-sufficient control mice (pOVA B6; Fig. 2 A). Of note, prolonged graft survival in pOVA µMT mice occurred in the presence or absence of serum reconstitution from naive mice (Fig. S1 C versus Fig. 2 A, respectively), indicating that prolonged graft survival was not due to an immunomodulatory effect of nonspecific antibody. Graft loss in rejecting pOVA µMT mice also occurred more slowly than in gOVA mice with kinetics similar to a primary allograft response (mean survival time, 13.2 ± 4.6 d [pOVA µMT] versus 14.6 ± 2.4 d [naive B6 + OVA skin graft]). In contrast, rapid OVA skin graft rejection was restored in pOVA μMT recipient mice that received serum from mice alloimmunized by a prior OVA skin graft or pregnancy (Fig. S1 B). Altogether, these experiments suggest that skin graft rejection can occur in parous mice, but despite alloimmunization and the presence of antigen-experienced CD8 T cells, the kinetics of this graft rejection is similar to that observed in naive mice in which T cells have not been primed.

**Figure S2.**
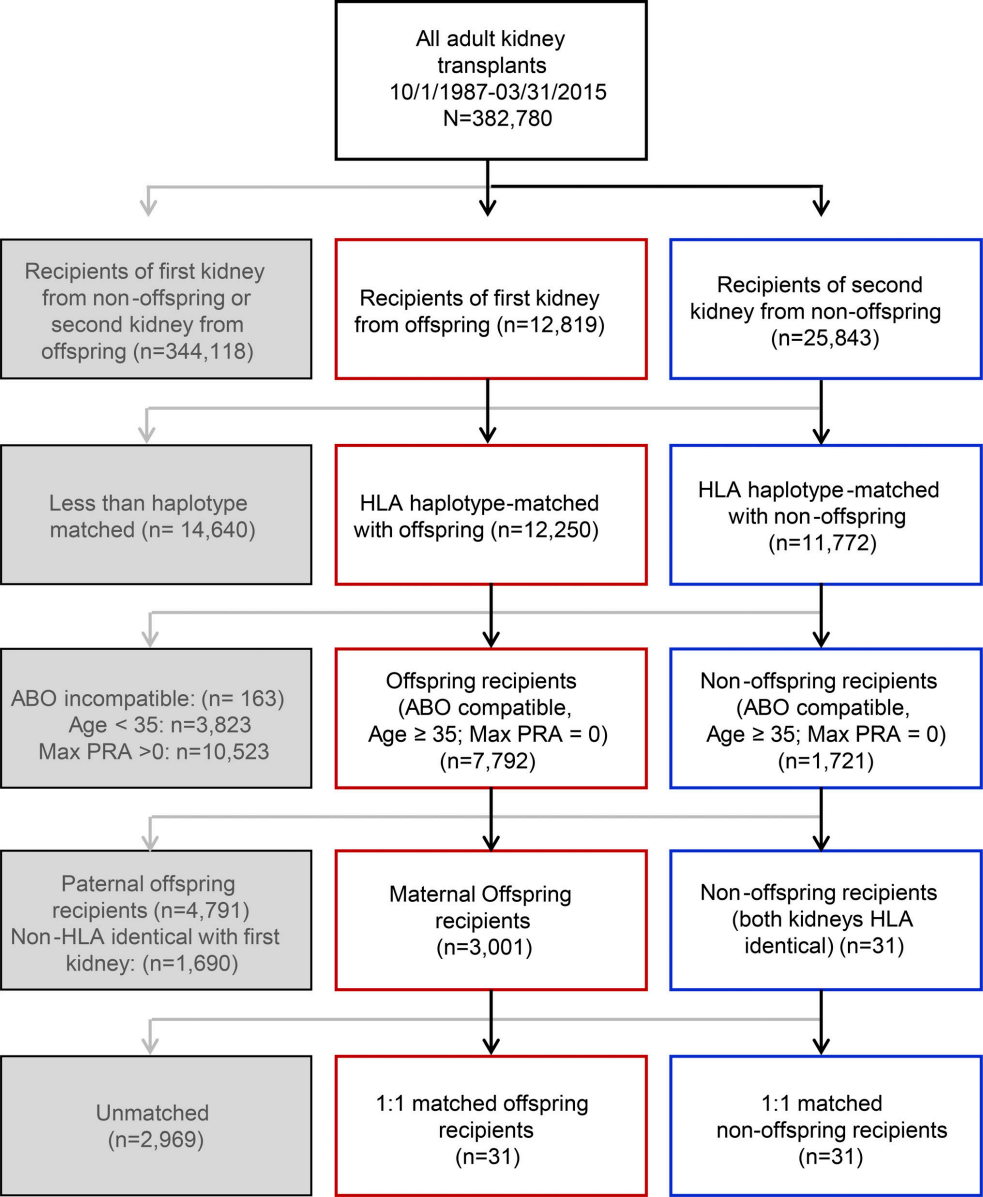
**Inclusion algorithm for 1:1 propensity score–matching analysis of kidney transplant recipients selected from the UNOS registry.** PRA, panel reactive antibody.

To investigate whether graft survival might be similarly prolonged in parous humans, we used registry data from the Organ Procurement and Transplantation Network (OPTN; *n* = 382,780 kidney transplant recipients) to compare kidney transplant allograft survival between female recipients of offspring kidneys and recipients of a second haplotype-matched kidney that was identical to the first kidney donor at the HLA-A, -B, and -DR loci (i.e., nonoffspring recipients; Fig. 2 B, Fig. S2, and Table S1). The cohorts were matched at these HLA loci because kidney donors and recipients are routinely typed at these loci, given the well-established role of these HLA loci in kidney transplant survival (Opelz and Döhler, 2007). Moreover, these loci are known to prime immune responses in pregnancy (Hönger et al., 2013). Recipients of a prior transplant (i.e., nonoffspring recipients) defined the expected event outcome as T_MEM_ cells have been demonstrated in these individuals and contribute to graft loss. Of note, we used propensity score matching to match patients from the offspring and nonoffspring cohorts who shared HLA specificity between their current transplant and their prior alloimmunization event. Consequently, the first alloimmunization event (either pregnancy or kidney transplant) was haplotype mismatched with the recipient, and the HLA specificity of the second alloimmunization event (i.e., kidney transplant in both recipient cohorts) was identical to the HLA specificity of the first alloimmunization event for both patient groups (Fig. 2 B, top; and Table S1).

There were 3,001 female recipients of offspring donor kidneys and 31 recipients of a second haplotype-matched kidney that was HLA identical with the first kidney (i.e., nonoffspring recipients). All individuals met inclusion criteria (Table S2), and 62 subjects achieved 1:1 matching. Using unadjusted Cox proportional hazards modeling in the matched cohort, there was a significantly increased risk of all-cause allograft failure among nonoffspring recipients compared with offspring recipients (Fig. 2 B; hazard ratio, 3.04; 95% confidence interval, 1.18–7.86). These data suggest that in parous humans, there is decreased allograft rejection compared with secondary allograft recipients. One interpretation of these data is that they reflect the scenario observed in mice above where CD8 T cell priming during pregnancy results in suboptimal T cell differentiation and lack of optimal T_MEM_ cells.

### Hypofunctional maternal CD8 T cells possess an exhaustion signature

Hypofunctional T cells arise in many contexts with different molecular mechanisms (Thommen and Schumacher, 2018; Schietinger and Greenberg, 2014; Gupta et al., 2019; Reiser and Banerjee, 2016; Kalekar and Mueller, 2017; Zheng et al., 2008; McLane et al., 2019; Pereira et al., 2017; Valdor and Macian, 2013; Chappert and Schwartz, 2010; Macián et al., 2002). Among these populations of hypofunctional T cells, exhaustion and anergy have been particularly well characterized. T cell exhaustion is a CD8 T cell fate that arises after the effector phase of an immune response during chronic viral infection and cancer. T_EX_ cells have high coexpression of inhibitory receptors (IRs) and a distinct transcriptional and epigenetic program (Wherry et al., 2007; Blackburn et al., 2009; Pauken et al., 2016; Sen et al., 2016; McLane et al., 2019). In contrast, anergic cells arise when costimulation is absent during priming, resulting in T cells that are never properly primed. Anergic T cells possess specific biochemical signaling defects and master transcription factors (Zheng et al., 2008; Chappert and Schwartz, 2010). We thus used these specific frameworks to gain additional insight into the differentiation state of hypofunctional maternal CD8 T cells.

**Figure 3.**
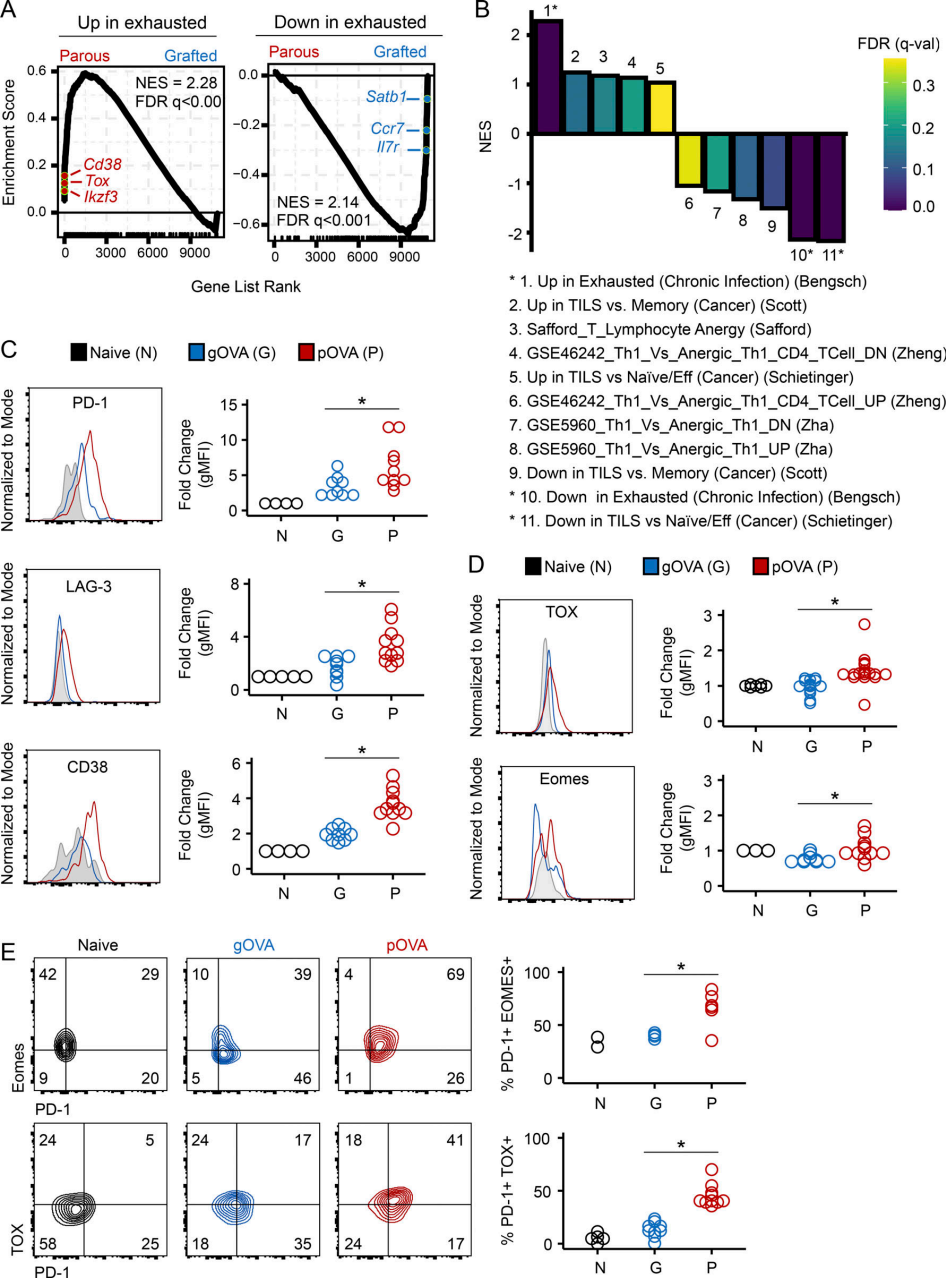
**Hypofunctional CD8 T cells in parous mice share defining features of exhaustion but not anergy.** **(A)** Enrichment of genes up- and down-regulated in T cell exhaustion (chronic viral infection; (Bengsch et al., 2018) in parous CD8 T cells. GSEA was performed using a gene expression dataset generated from bulk RNA-seq of OT-1 T cells sorted from naive^OT-1^, pOVA^OT-1^, and gOVA^OT-1^ mice. FDR, false discovery rate; NES, normalized enrichment score. Select leading edge genes are depicted. **(B)** Summary of GSEA performed between OT-1 T cell expression dataset and anergic and exhausted gene sets. Asterisks indicate FDR < 0.05. Eff, T effector cells; TILS, tumor-infiltrating lymphocytes. **(C)** IR expression on OT-1 cells. OT-1 cells from naive^OT-1^, pOVA^OT-1^, and gOVA^OT-1^ mice were analyzed 50–60 d after cell transfer. Histograms are gated on congenic OT-1 T cells; undivided CD44^−^ cells are gated in naive mice, while divided CD44^+^ cells are gated in pOVA and gOVA mice. Data are pooled from two experiments. Naive^OT-1^, *n* = 4–5; gOVA^OT-1^, *n* = 9; pOVA^OT-1^, *n* = 10–11. PD-1, *, P = 0.02; LAG-3, *, P = 0.004; CD38, *, P < 0.001. Student’s *t* test. **(D)** OT-1 cells in pOVA mice express transcription factors associated with T cell exhaustion. Data are pooled from two experiments. Naive^OT-1^, *n* = 3–7; gOVA^OT-1^, *n* = 9–15; pOVA^OT-1^, *n* = 11–19. TOX, *, P = 0.02; Eomes, *, P = 0.01. Student’s *t* test. **(E)** Coexpression of IRs and transcription factors associated with T cell exhaustion. Data are pooled from two experiments. Naive^OT-1^, *n* = 2–5; gOVA^OT-1^, *n* = 4–9; pOVA^OT-1^, *n* = 6–10. %PD-1+ Eomes+, *, P = 0.01; %PD-1+ Tox+, *, P < 0.001.

We first asked whether gene sets associated with T cell exhaustion or anergy were enriched in the maternal OVA-specific CD8 T cell transcriptome. To identify the genes associated with pregnancy-induced T cell hypofunction, we performed whole-genome transcriptional analysis of OT-1 cells (i.e., RNA sequencing [RNA-seq]) from gOVA^OT-1^, pOVA^OT-1^, and naive^OT-1^ mice. We ranked genes differentially expressed in OT-1 cells from pOVA^OT-1^ versus gOVA^OT-1^ mice and performed gene set enrichment analysis (GSEA) using benchmark exhaustion (Bengsch et al., 2018; Scott et al., 2019; Schietinger et al., 2016) or anergy gene sets (Safford et al., 2005; Zheng et al., 2013; Zha et al., 2006). We found significant enrichment of genes up- and down-regulated in exhaustion in parous mice, with the strongest enrichment of gene sets derived from mouse models of chronic infection (Fig. 3, A and B). Leading edge genes included highly differentially expressed genes in parous versus grafted OT-1 cells, including *Tox*,* Ikzf3*,* Cd38*,* Satb1*, and *Rpl17*, among others (Fig. 3 A). Conversely, no significant enrichment of anergy gene sets was observed in our expression dataset (Fig. 3 B). Together, these studies suggested that hypofunctional maternal CD8 T cells in parous mice were transcriptionally similar to T_EX_ cells.

Next, we evaluated markers of differentiation state and biochemical features to gain further insights into the developmental biology of OT-1 T cells primed during pregnancy. Although IRs are expressed by activated T cells (Morris et al., 2018), sustained expression of multiple IRs together is a hallmark of T_EX_ (Blackburn et al., 2009; McLane et al., 2019), and blockade of IRs can improve T cell functionality (Barber et al., 2006; Pauken et al., 2016). Indeed, expression of multiple IRs was increased by OT-1 cells isolated from pOVA^OT-1^ mice compared with gOVA^OT-1^ mice, including PD-1 and LAG-3 (Fig. 3 C). OT-1 T cells from parous mice also expressed higher amounts of transcription factors associated with T cell exhaustion, including Eomesodermin (Eomes) and TOX (Fig. 3 D; Khan et al., 2019; Scott et al., 2019; Alfei et al., 2019; Seo et al., 2019; Paley et al., 2012; Doering et al., 2012) both of which were coexpressed with PD-1 (Fig. 3 E; Khan et al., 2019; Scott et al., 2019; Alfei et al., 2019; Paley et al., 2012). In contrast, elevated expression of molecular mediators of anergy (e.g., Egr-2 and Cbl-b; Harris et al., 2004; Safford et al., 2005; Zheng et al., 2013; Zha et al., 2006; Zheng et al., 2012; Mueller, 2004; Jeon et al., 2004) was not detected in any group. Specifically, Cbl-b expression in OT-1 cells from both gOVA and pOVA groups was similar to OT-1 T cells in naive mice (105 ± 18% of naive [pOVA] versus 111 ± 6% of naive [gOVA], P = ns). Egr-2 expression in OT-1 cells from both gOVA and pOVA mice was also similar, with mildly reduced expression compared with OT-1 cells from naive mice (62 ± 22% of naive [pOVA] versus 83 ± 28% of naive [gOVA], P = ns). Finally, we considered whether signaling pathways induced in T cell anergy were used by OT-1 T cells from parous mice. Because anergic T cells have diminished capacity to phosphorylate ERK (Fields et al., 1996; Dillon et al., 2003), we examined ERK phosphorylation in unstimulated and antigen-stimulated OT-1 T cells recovered from gOVA^OT-1^, pOVA^OT-1^, and naive^OT-1^ mice. OVA peptide stimulation induced phosphorylation of ERK in all OT-1 T cells, with no appreciable difference in phosphorylated ERK in OT-1 cells from pOVA^OT-1^ or gOVA^OT-1^ mice (fold change in phosphorylated ERK versus unstimulated naive cells: OT-1 from pOVA^OT-1^ = 1.22 versus gOVA^OT-1^ = 1.26; P = ns). Collectively, these data suggested that pregnancy induces a state of T cell differentiation similar to exhaustion rather than anergy in fetal-specific maternal CD8 T cells.

### Pregnancy induces an attenuated exhaustion state compared with chronic viral infection

**Figure 4.**
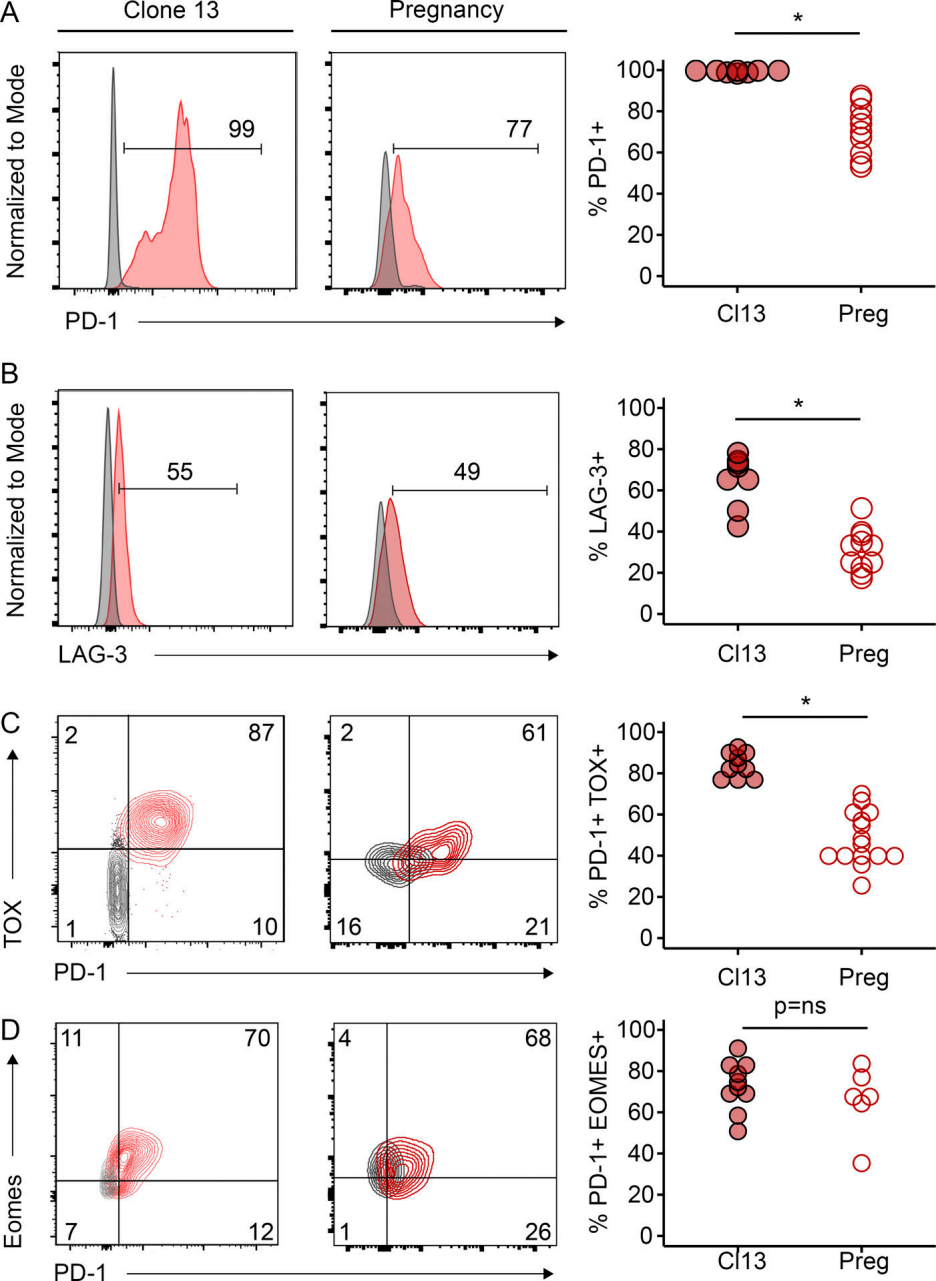
**Pregnancy induces an attenuated exhaustion phenotype compared with chronic viral infection.** PD-1, LAG-3, TOX, and Eomes expression was assessed on either P14 LCMV-specific CD8 T cells in clone 13 (Cl13)–infected mice (left column) or on OT-1 OVA-specific CD8 T cells in Act-mOVA–mated parous mice (middle column). For LCMV-infected mice, 1,000 P14 congenic CD8 T cells were adoptively transferred 1 d before i.v. infection with LCMV clone 13. P14 cells were recovered from the spleens of infected mice ∼30 d after infection. Histograms and flow plots are included from representative mice and are gated on congenic P14 cells. Gray histograms represent protein expression on naive CD44^−^ CD8 T cells within the endogenous repertoire. For pregnancy (Preg) experiments, 3 × 10^6^ congenic CTV-labeled OT-1 T cells were adoptively transferred into B6 mice that were then exposed to OVA antigen through pregnancy immunization (as in Fig. 1 A). 50–60 d after transfer, protein expression on OT-1 CD8 T cells in the spleen was assessed using flow cytometry. Flow plots and histograms are included from representative mice. Data in gray represent protein expression on undivided OT-1 T cells from naive mice without OVA exposure, while data represented in red derive from divided OT-1 T cells from OVA-parous mice. Circles in scatter plots represent individual mice. **(A)** Clone 13, *n* = 8, pooled from two independent experiments; pregnancy, *n* = 10, pooled from two independent experiments. *, P < 0.001. Student’s *t* test. **(B)** Clone 13, *n* = 8, pooled from two independent experiments; pregnancy, *n* = 11, pooled from two independent experiments. *, P < 0.001. Student’s *t* test. **(C)** Clone 13, *n* = 10, pooled from two independent experiments; pregnancy, *n* = 15, pooled from two independent experiments. *, P < 0.001. Student’s *t* test. **(D)** Clone 13, *n* = 10, pooled from two independent experiments; pregnancy, *n* = 6, one experiment is shown.

We next further explored the similarities between pregnancy-induced T_EX_ cells (Preg-T_EX_) and T_EX_ cells from other settings. We therefore compared Preg-T_EX_ and T_EX_ cells from lymphocytic choriomeningitis virus (LCMV) clone 13–infected mice. 10^3^ TCR transgenic P14 T cells specific for the H2-D^b^-restricted gp33-41 epitope of LCMV were adoptively transferred into C57BL/6 mice that were infected with LCMV clone 13. Expression of PD-1, LAG-3, TOX, and Eomes was assessed in P14 cells recovered from the spleens of infected animals ∼30 d after infection. With the exception of Eomes, chronic viral infection induced higher expression of these key markers of exhaustion than pregnancy (Fig. 4). Thus, direct comparison with T_EX_ cells from a canonical model of CD8 T cell exhaustion suggests that the exhaustion that may occur for Preg-T_EX_ cells may be less severe or less robust, at least based on lower expression of these key molecules.

### Maternal CD8 T cells have reduced expression of protein translation machinery

**Figure 5.**
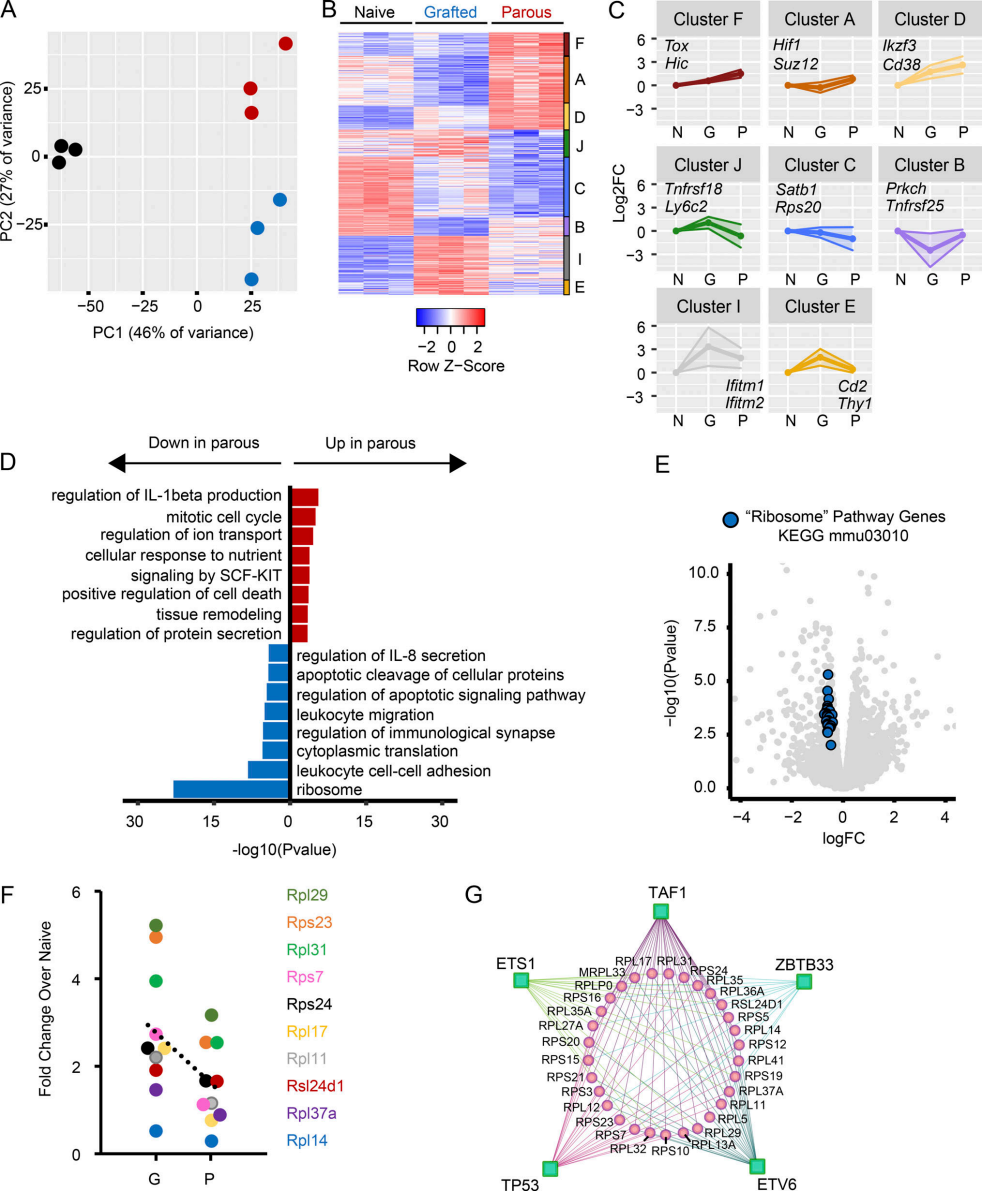
**The transcriptome of hypofunctional maternal T cells reveals translational repression.** Naive^OT-1^, gOVA^OT-1^, and pOVA^OT-1^ were generated as in Fig. 1 A. 50–60 d after OT-1 transfer, OT-1 cells were sorted from secondary lymphoid organs and analyzed with bulk RNA-seq. 10 mice were pooled per sample; 3 samples per group were sequenced as shown. **(A)** Principal component (PC) analysis plot of gene expression. **(B)** Heat map of 400 differentially expressed genes (DEGs) in OT-1 T cells (DEGs = pOVA versus gOVA; FDR q < 0.05). Individual clusters are shown. **(C)** Line graphs of gene expression (log_2_ fold change [Log_2_FC]) in each DEG cluster. Select representative genes from each cluster are shown. Data points represent average expression. Shaded areas represent SD. N, naive; G, grafted; P, parous. **(D)** Biological pathways up-regulated and down-regulated in pOVA mice. SCF, stem cell factor. **(E)** Volcano plot of all expressed genes (gray) sequenced in naive, gOVA, and pOVA animals. Blue circles represent 30 ribosomal protein genes down-regulated in the Ribosome pathway (from KEGG: mmu03010). **(F)** RT-PCR results confirming reduced expression of genes encoding select ribonucleic proteins in OVA-parous mice. **(G)** Predicted regulon of ribosomal protein genes down-regulated in parous CD8 T cells. Green squares, transcriptional regulators; pink circles, ribosomal protein genes.

To better understand the molecular mechanisms underlying pregnancy-induced T cell exhaustion, we further examined the transcriptomes of OT-1 cells from gOVA^OT-1^, pOVA^OT-1^, and naive^OT-1^ mice. Bulk RNA-seq revealed transcriptomic differences between the groups (Fig. 5 A) and identified 400 differentially expressed genes between OT-1 cells in pOVA^OT-1^ versus gOVA^OT-1^ mice (Fig. 5 B and Table S3). We validated key differential gene and protein expression differences by quantitative PCR and flow cytometry and found that *Tox*, *Cd38*, and *Ikzf3* were among genes up-regulated in parous mice, whereas *Satb1* was down-regulated.

The RNA-seq analysis also revealed that numerous genes encoding ribosomal proteins were down-regulated in Preg-T_EX_ cells (i.e., *Rpl12*,* Rps20*, etc.) compared with T_MEM_ cells from gOVA mice. Accordingly, biological pathways associated with the ribosome and cytoplasmic protein translation were significantly down-regulated in Preg-T_EX_ cells (Fig. 5 C, bottom). These pathways were largely enriched because of transcriptional down-regulation of 32 coregulated ribosomal proteins (Fig. 5 D), suggesting that pregnancy restricts T cell function by limiting the ribosomal translational machinery. Several of these gene expression changes were validated by RT-PCR (Fig. 5 E). Coregulation of ribosomal proteins is highly conserved across species (Ishii et al., 2006; Li et al., 2016; Moreira-Ramos et al., 2018), allowing this information to be used to predict the transcriptional regulators that impact the ribosomal protein network. This analysis revealed *Ets1* and *Tp53* among 10 other transcription factors (Fig. 5 F) that are known to control cell division, quiescence, and metabolism in other settings (Golomb et al., 2014; Garrett-Sinha et al., 2016) as potential regulators of exhaustion in Preg-T_EX_ cells. Altogether, these data identify repression of the protein translation machinery as a key aspect of the exhaustion program employed by Preg-T_EX_ cells.

### NFAT signals down-regulate protein translation across different exhaustion contexts

**Figure 6.**
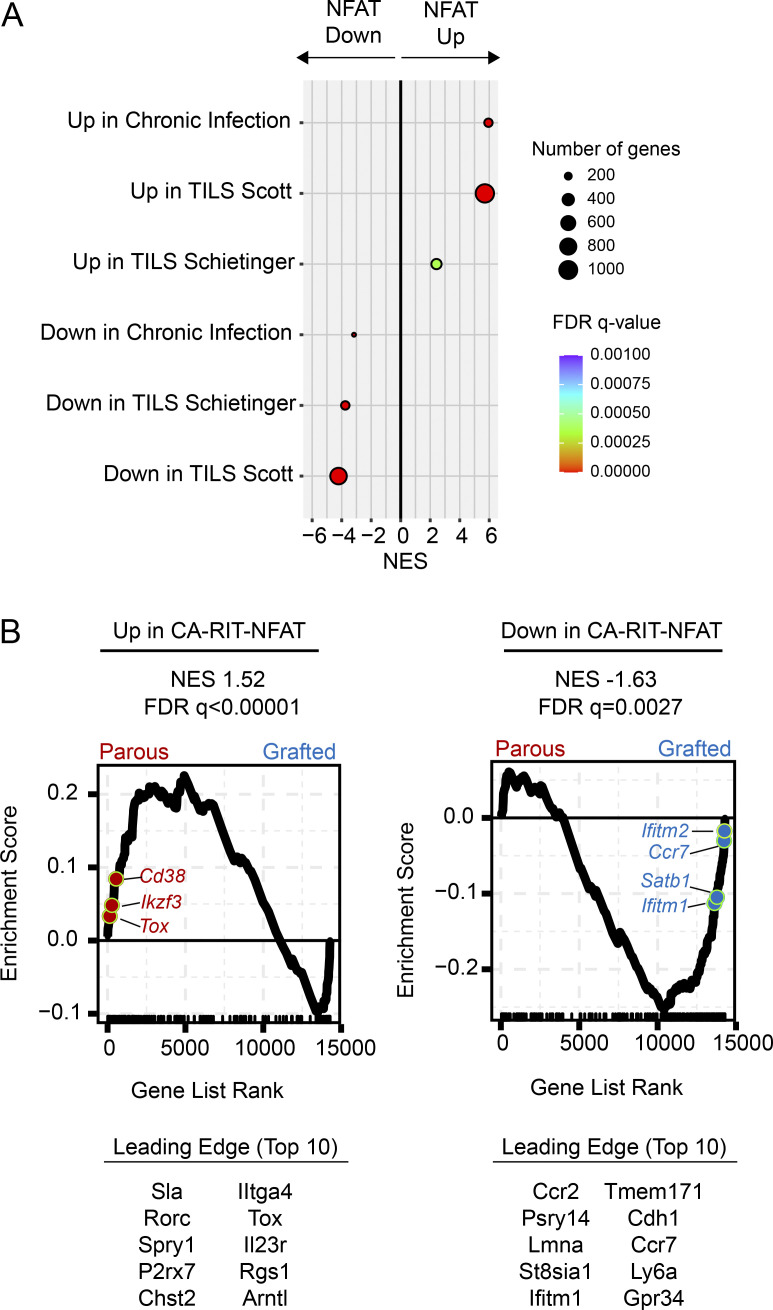
**The gene signature induced by partnerless NFAT is shared across exhaustion contexts.** Naive^OT-1^, gOVA^OT-1^, and pOVA^OT-1^ were generated as in Fig. 1 A. 50–60 d after OT-1 transfer, OT-1 cells were sorted from secondary lymphoid organs and analyzed with bulk RNA-seq as in Fig. 5. The CA-RIT-NFAT1 gene signature was derived from Martinez et al. (2015). **(A)** Enrichment of exhaustion gene sets in the CA-RIT-NFAT1 signature. FDR, false discovery rate; NES, normalized enrichment score; TILS, tumor-infiltrating lymphocytes. **(B)** Enrichment of CA-RIT-NFAT1 gene signature in Preg-T_EX_ cells. (Schietinger et al., 2016; Scott et al., 2019).

Down-regulation of genes encoding ribosomal subunits has long been known to be a key transcriptional feature of exhaustion (Wherry et al., 2007). Given the importance of NFAT as a driver of T cell exhaustion (Martinez et al., 2015; Seo et al., 2019; Khan et al., 2019; Scott et al., 2019), we next asked whether NFAT might contribute to this translational repression. To identify the biological processes controlled by NFAT in hypofunctional T cells, we analyzed gene sets derived from T cells that were transduced with a constitutively active form of NFAT that cannot partner with AP-1 (CA-RIT-NFAT1; Martinez et al., 2015). So-called partnerless NFAT has been shown to promote a program of gene expression changes that are enriched in T_EX_ cells (Martinez et al., 2015). We first confirmed that T_EX_ cells induced by chronic viral infection or cancer had transcriptional imprints of partnerless NFAT activity by testing whether genes up- and down-regulated by CA-RIT-NFAT1 were also enriched in these contexts. Transcriptional signatures from T_EX_ cells displayed clear overlap with the transcriptional signatures induced by CA-RIT-NFAT1 (Fig. 6 A). We then evaluated whether the CA-RIT-NFAT1 signature was similarly enriched in OT-1 T cells from parous mice. Indeed, strong enrichment of genes up- and down-regulated by CA-RIT-NFAT1 was found in OT-1 cells primed during pregnancy (Fig. 6 B).

**Figure 7.**
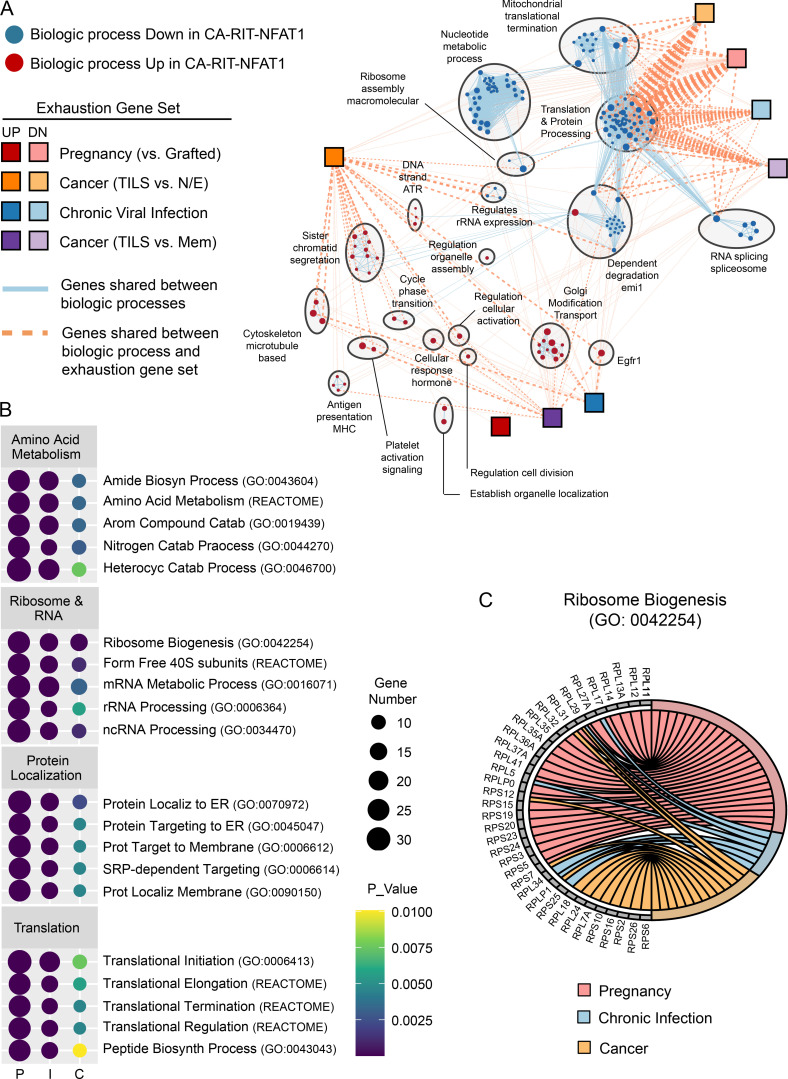
**Translational repression is a conserved transcriptional mechanism of exhaustion induced by NFAT.** **(A)** Exhaustion gene sets (squares) are superimposed on a network of biological processes that are up-regulated (red circles) or down-regulated (blue circles) by partnerless NFAT (CA-RIT-NFAT1; Martinez et al., 2015). Biological processes are clustered based on gene sharing (light gray circles). Red or blue circle size maps to normalized enrichment score (NES) of biological process gene set in CA-RIT-NFAT1 expression dataset. Blue line size reflects gene overlap between biological processes. Orange line size reflects gene overlap between a biological process gene set and an exhaustion gene set. TILS, tumor-infiltrating lymphocytes.** (B)** Enrichment of exhaustion gene sets within selected biological processes of the translation and protein processing cluster. I, chronic viral infection; P, pregnancy (Bengsch et al., 2018); C, cancer (Schietinger et al., 2016). **(C)** Ribosomal protein genes from Ribosome Biogenesis (Gene Ontology term 0042254) are down-regulated by NFAT and shared between exhaustion gene sets. N/E, naive/effector.

Next, we created a network of the biological processes that were significantly influenced by CA-RIT-NFAT1 (Fig. 7 A) by performing GSEA of the CA-RIT-NFAT1 expression dataset with gene sets representing all known biological processes (*n* = 15,736; see Materials and methods). Biological processes were clustered and annotated using EnrichmentMap (Reimand et al., 2019; Merico et al., 2010), and exhaustion gene sets were superimposed onto this network. This analysis revealed 183 biological processes that were significantly up- or down-regulated by CA-RIT-NFAT1. Genes governing the majority of these processes (*n* = 140; 76%) were down-regulated by partnerless NFAT, with a predominance of down-regulated gene sets governing protein translation (translation and protein processing cluster; 40% of down-regulated gene sets [56 of 140]). Notably, there was significant overlap between down-regulation of protein translation by NFAT and gene sets down-regulated in Preg-T_EX_ cells as well as T_EX_ cells in chronic viral infection and cancer (Fig. 7 A). This overlap was driven largely by ribosomal protein genes that were down-regulated in T cell exhaustion and included in numerous biological processes associated with protein translation (Fig. 7, B and C). Collectively, these analyses suggested that the down-regulation of cellular translational machinery by NFAT is a conserved molecular mechanism of T cell exhaustion.

### Exhaustion programming of maternal T cells depends on NFAT and fetal antigen

**Figure 8.**
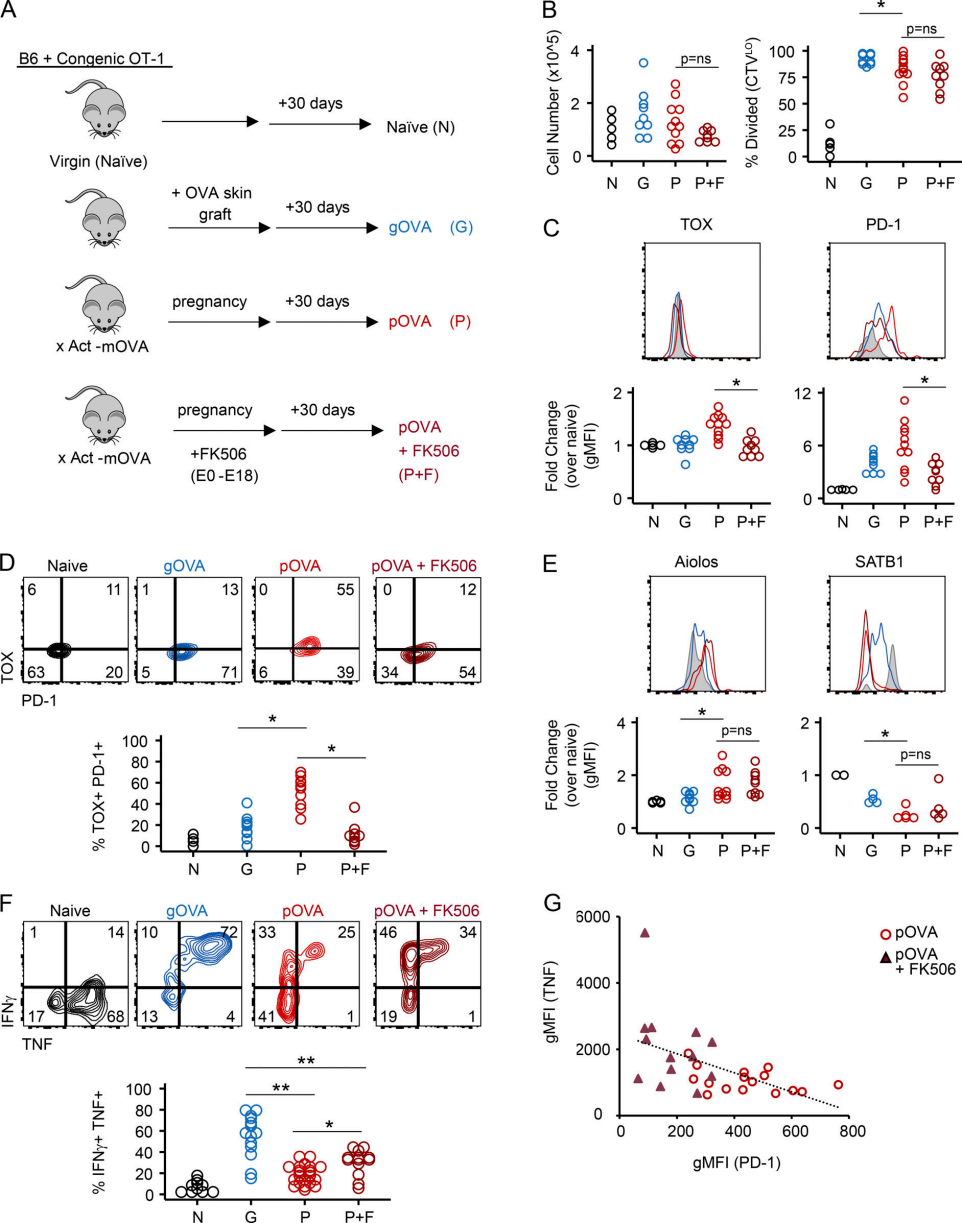
**FK506 treatment reveals NFAT-dependent and –independent elements of the pregnancy exhaustion program.** **(A)** Schematic of experimental design. 3 × 10^6^ CD45.2 OT-1 T cells were adoptively transferred into CD45.1 mice. pOVA^OT-1^ were generated by mating with Act-mOVA males. Control pOVA^OT-1^ mice (P) were injected with PBS, while another group of pOVA^OT-1^ mice was injected with FK506 (1 mg/kg i.p. daily) from E0.5–E18.5 mice during pregnancy (P+F). naïve^OT-1^ (N) and gOVA^OT-1^ (G) mice were generated as in Fig. 1 A. Spleens were analyzed 50–60 d after transfer of OT-1 cells. **(B)** Equivalent number of OT-1 cells and similar frequency of divided OT-1 T cells in pOVA^OT-1^ versus FK506-treated pOVA^OT-1^ mice. Data are pooled from two experiments N, *n* = 5; G, *n* = 9; P, *n* = 11; P+FK506, *n* = 9. *, P < 0.04; Student’s *t* test. **(C and D)** NFAT blockade during pregnancy prevents induction of TOX and PD-1 on OT-1 cells. OT-1 cells from naive^OT-1^ mice are gated on CTV^hi^ CD44^−^, while OT-1 cells from pOVA^OT-1^ mice are gated on CTV^Lo^ divided CD44^+^ cells. Data are pooled from two experiments. N, *n* = 5; G, *n* = 9; P, *n* = 11; P+FK506, *n* = 9. TOX, *, P < 0.001. PD-1, *, P = 0.004; Student’s *t* test. gMFI, geometric mean fluorescence intensity. **(E)** Aiolos and SATB1 in OT-1 cells isolated from pOVA^OT-1^ mice are not altered by FK506 treatment. Aiolos data are pooled from two experiments. N, *n* = 5; G, *n* = 9; P, *n* = 11; P+FK506, *n* = 9. Data for SATB1 are from a single experiment. N, *n* = 2; G, *n* = 4; P, *n* = 5; P+FK506, *n* = 5. *, P = 0.02; Student’s *t* test. **(F)** Nominal rescue of cytokine production by FK506 treatment of pOVA^OT-1^ mice. Splenocytes were stimulated with OVA peptide (SIINFEKL) directly ex vivo. Data are pooled from four experiments. N, *n* = 10; G, *n* = 14; P, *n* = 22; P+FK506, *n* = 13. *, P < 0.01; **, P < 0.001; Student’s *t* test. **(G)** Correlation of PD-1 and TNF expression between pOVA^OT-1^ and FK506-treated pOVA^OT-1^ mice. Pooled from three experiments. P, *n* = 16; P+FK506, *n* = 13.

CD8 T cell exhaustion is initiated by continuous NFAT activity early after antigen encounter through induction of the high-mobility group transcription factor TOX (Khan et al., 2019; Scott et al., 2019; Alfei et al., 2019; Seo et al., 2019). We hypothesized that fetal antigen induced CD8 T cell exhaustion in pregnancy through a similar molecular circuit. To test whether NFAT was required for TOX and IR expression in Preg-T_EX_ cells, we treated OVA-mated mice with either PBS or the NFAT inhibitor FK506 during pregnancy and analyzed OT-1 T cells 50–60 d after cell transfer (Fig. 8 A). Control cohorts of naive^OT-1^ and gOVA^OT-1^ mice were included for comparison. Given the ability of FK506 to limit T cell proliferation and effector function at high doses (Tocci et al., 1989; Hu et al., 2003), we selected a low dose of FK506 (1 mg/kg). At this dose, an equivalent number of OT-1 cells were recovered from FK506- and PBS-treated pOVA^OT-1^ mice, and a similar percentage of these cells had undergone division (Fig. 8 B). Consistent with the effects of FK506 on T_EX_ cells in cancer and chronic infection (Khan et al., 2019; Scott et al., 2019), FK506 diminished the coexpression of TOX and PD-1 (Fig. 8, C and D). Whereas TOX and PD-1 expression were reduced at this dose of FK506, other transcription factors altered by Preg-T_EX_ cells and T_EX_ cells in cancer and chronic infection compared with canonical memory T cells (i.e., Aiolos [*Ikzf3*], SATB1) were less impacted by FK506 treatment, indicating a selective effect on some but not all pathways of T cell differentiation (Fig. 8 E). Treatment with FK506 increased cytokine production from Preg-T_EX_ cells, though this function remained inferior to that of T_MEM_ cells in gOVA^OT-1^ mice (Fig. 8 F). We next examined the correlation of PD-1 expression with TNF production in Preg-T_EX_ cells recovered from PBS-treated and FK506-treated parous mice (Fig. 8 G). Although there was a modest inverse correlation between PD-1 and TNF expression (r = −0.53; P < 0.01), large changes in PD-1 expression were only associated with small changes in TNF. Moreover, the coefficient of determination was low (R^2^ = 0.2784), suggesting that PD-1 expression was a poor predictor of TNF expression. These results suggested that the TOX–PD-1 axis was decoupled to some extent from T cell function, as has been observed in other T_EX_ cells (Scott et al., 2019; Khan et al., 2019). Thus, these experiments not only confirm the dependency of TOX and PD-1 expression on NFAT signals but also reveal a role for the NFAT circuit in Preg-T_EX_ cells.

**Figure 9.**
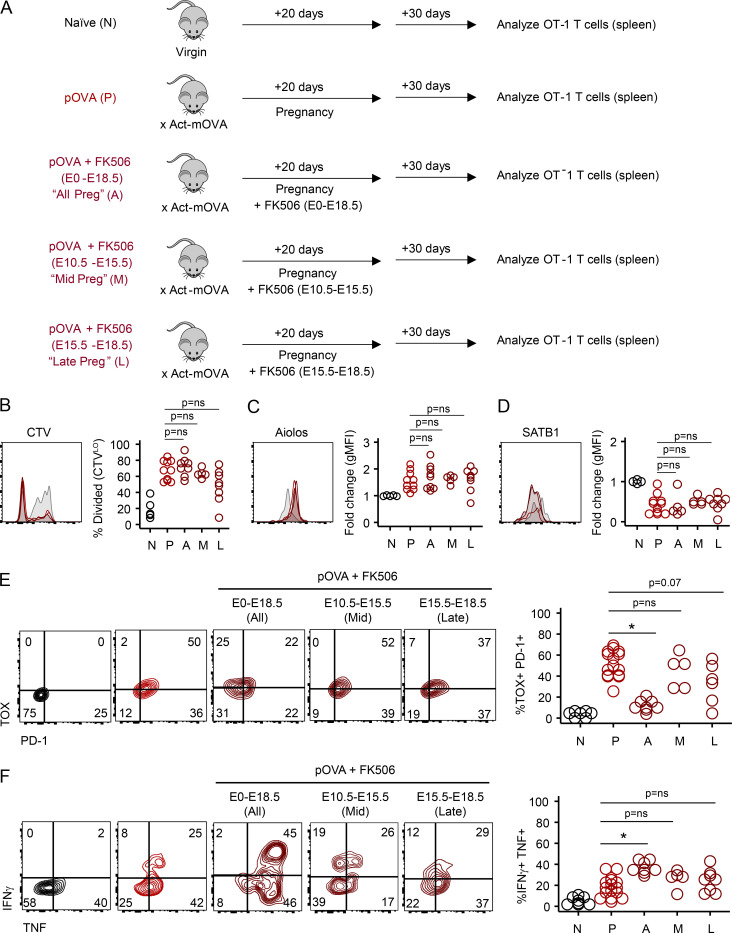
**NFAT-dependent elements of the maternal T cell exhaustion program are induced by fetal antigen during pregnancy. (A)** Schematic of experimental design. Naive^OT-1^ and pOVA^OT-1^ mice were generated as in Fig. 1 A. Groups of pOVA^OT-1^ mice received either PBS injection or FK506 (1 mg/kg i.p.) at different times during gestation. OT-1 cells recovered from the maternal spleen were analyzed 30 d after pups were born (50 d after transfer of OT-1 cells). Flow plots are gated on OT-1 T cells from representative mice. Gray histograms = expression in naive mice. For clarity in B–D, only the overlays from pOVA (red) and two FK506 treatment groups (pOVA + FK506 E15.5–E18.5 [dark red shaded] and pOVA + FK506 E10.5–E15.5 [dark red]) are shown. Data are pooled from three experiments. N, *n* = 5; P, *n* = 10; P+FK506 (E0.5-E18.5), *n* = 8; P+FK506 (E10.5–E15.5), *n* = 5; P+FK506 (E15.5–E18.5), *n* = 8. **(B)** Similar cell division between pOVA^OT-1^ mice and FK506-treated pOVA^OT-1^ mice. Student’s *t* test. **(C and D)** NFAT-independent elements (i.e., Aiolos, SATB1) of the exhaustion program are unaffected by FK506 treatment at any time during gestation. Student’s *t* test. gMFI, geometric mean fluorescence intensity. **(E)** Expression of NFAT-dependent elements PD-1 and TOX is driven by antigen exposure primarily in late gestation. *, P < 0.001; Student’s *t* test. **(F)** Nominal rescue of cytokine production by FK506 blockade is driven by antigen exposure throughout pregnancy and correlates with TOX:PD1 expression. *, P < 0.001; Student’s *t* test.

In cancer and chronic infection, TOX is expressed downstream of persistent NFAT signals driven by chronic antigen stimulation. To test the hypothesis that fetal antigen was a similar driver of TOX expression in Preg-T_EX_ cells, we interrupted antigen signals at various time points during pregnancy with FK506 treatment and assessed T cell exhaustion 30 d postpartum (Fig. 9 A). Fetal antigen initially becomes systemically available to prime maternal T cells at approximately embryonic day 10 (E10) and escalates throughout the course of gestation (Erlebacher et al., 2007; Moldenhauer et al., 2009; Tay et al., 2013). We therefore selected time points for FK506 treatment that coincided with differences in antigen dose in the spleen. As above, treatment of pregnant mice with this low dose of FK506 did not significantly impair CD8 T cell proliferation (Fig. 9 B), nor did it alter Aiolos up-regulation or SATB1 down-regulation in Preg-T_EX_ cells (Fig. 9, C and D). In contrast to mice treated with FK506 throughout pregnancy, however, transient disruption of NFAT signaling in middle or late pregnancy did not significantly reduce TOX and PD-1 expression (Fig. 9 E). These data suggest that even brief periods of NFAT signaling were thus sufficient to promote TOX and PD-1 up-regulation. Although TOX and PD-1 were induced by antigen exposure in either early or late pregnancy, exposure to antigen late in pregnancy appeared to be particularly influential, given that TOX and PD-1 expression was lowest in mice receiving FK506 late in pregnancy (i.e., FK506 treatment E15.5–E18.5) and highest in mice exposed to antigen after E15.5, as occurred in the E10.5–E15.5 treatment group. Although PD-1 and TOX expression may be influenced by NFAT signals that occur before E10.5 in the mid-pregnancy FK506 treatment group, increases in PD-1 and TOX expression in this group are more likely due to exposure to antigen in late pregnancy (>E15.5), given limited antigen availability in the spleen before E10.5 (Erlebacher et al., 2007; Moldenhauer et al., 2009). Altogether, these data demonstrate that TOX and PD-1 expression in pregnancy is NFAT dependent. In contrast, the NFAT-independent elements of Preg-T_EX_ cells (i.e., SATB1, Aiolos) were induced despite FK506 treatment (Fig. 9, C and D).

T cell function was impacted by both NFAT-dependent and -independent elements of the pregnancy exhaustion program. Although treatment with FK506 throughout the entirety of gestation resulted in a marginal increase in cytokine production (Fig. 9 F), NFAT blockade was insufficient to restore maternal T cell functionality to the level observed in T_MEM_ cells from gOVA^OT-1^ mice (60–80%; Figs. 1 F and 8 F). Nevertheless, tuning of cytokine production through the NFAT/TOX/PD-1 pathway appeared to track with longer duration of antigen exposure in pregnancy, because treatment with FK506 for the full period of gestation was necessary for even the marginal boost in cytokine production. Thus, these experiments reveal a role for both NFAT-dependent and likely NFAT-independent components of the pregnancy-induced CD8 T cell exhaustion program.

### Evidence of exhaustion programming of maternal CD8 T cells in the primary effector phase

**Figure 10.**
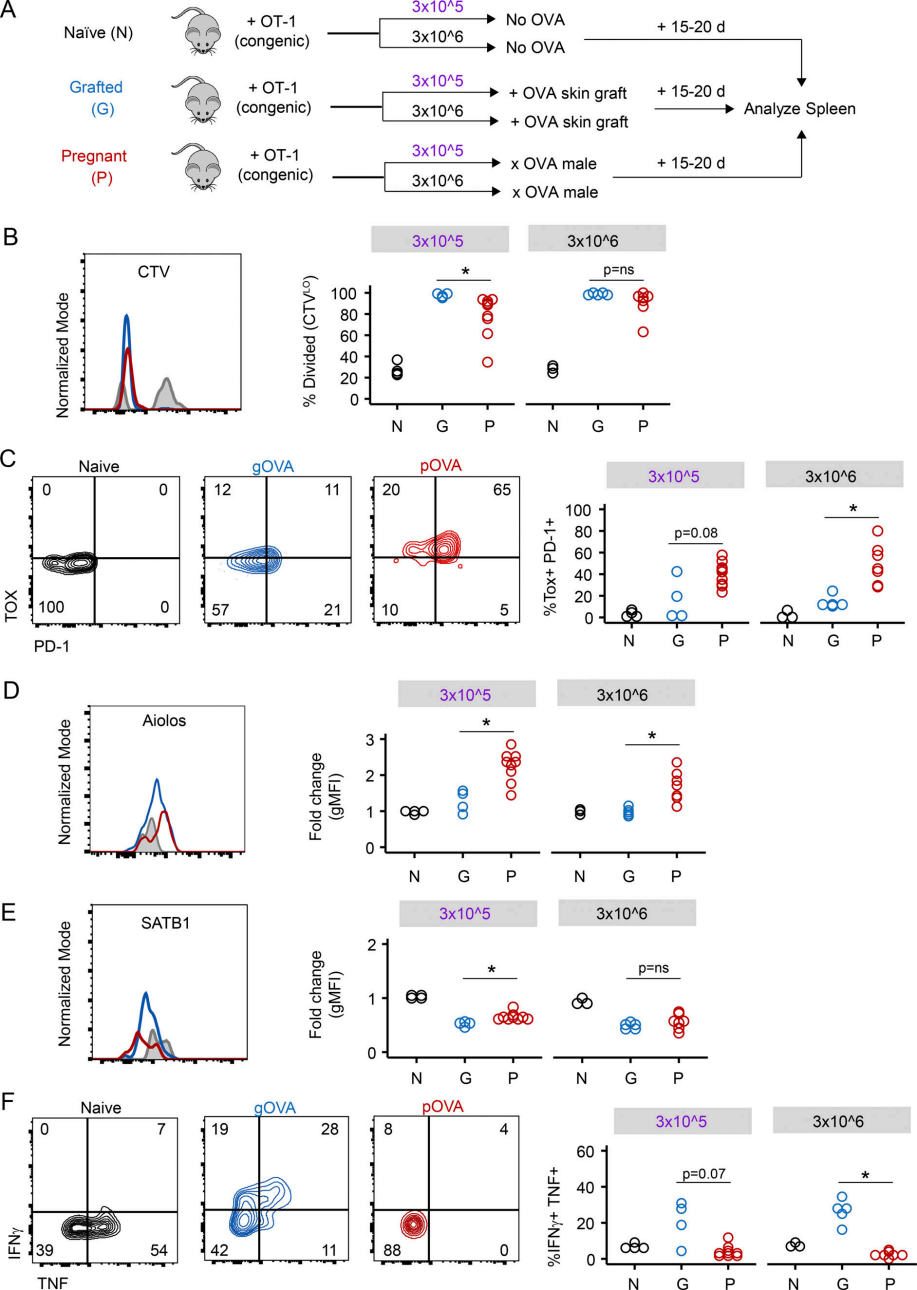
**Exhaustion programming of maternal CD8 T cells occurs during primary effector differentiation.** **(A)** Schematic of experimental design. 3 × 10^5^ or 3 × 10^6^ CTV-labeled CD45.2 OT-1 T cells were adoptively transferred into CD45.1 congenic mice. Mice received no OVA exposure (Naive^OT-1^ [N]), received an OVA skin graft (gOVA^OT-1^ [G]), or were mated with an Act-mOVA male (pOVA^OT-1^ [P]). Spleens were analyzed on days 15–20 after OT-1 cell transfer. Flow plots and histograms are gated on OT-1 CD8 T cells and are representative of individual mice within each group. With the exception of B, all plots are additionally gated on CTV^Hi^ undivided cells (Naive^OT-1^ [black or gray]) or gated on CTV^Lo^ divided cells (gOVA^OT-1^ [blue], pOVA^OT-1^ [red]). Data are pooled from two experiments. Circles represent individual mice; Naive^OT-1^, *n* = 7; gOVA^OT-1^, *n* = 9; pOVA^OT-1^, *n* = 16. **(B)** Primary effector OT-1 T cells divide early after OVA antigen exposure. *, P = 0.02; Student’s *t* test. **(C)** TOX and PD-1 expression are increased during primary effector differentiation in pOVA^OT-1^ mice. *, P < 0.002; Student’s *t* test. **(D and E)** Aiolos and SATB1 expression in primary effector OT-1 T cells and antigen-experienced populations. Aiolos, *, P < 0.002 (3 × 10^5^); *, P < 0.004 (3 × 10^6^); SATB1, *, P < 0.003 (3 × 10^5^); Student’s *t* test. gMFI, geometric mean fluorescence intensity. **(F)** Lack of cytokine production by primary effector OT-1 CD8 T cells in pOVA^OT-1^ mice. *, P < 0.001; Student’s *t* test.

We next asked when elements of exhaustion programming manifested during pregnancy. We therefore adoptively transferred 3 × 10^6^ or 3 × 10^5^ CTV-labeled OT-1 cells into congenic mice that (1) received no OVA exposure, (2) received an OVA skin graft, or (3) were mated with an Act-mOVA male (Fig. 10 A). In contrast to the experiments above that examined T cell differentiation after ∼1–2 mo, here we examined T cell activation and differentiation at E15.5–E18.5. OVA antigen first becomes systemically available to prime maternal T cells at approximately E10.5 (Erlebacher et al., 2007). Thus, examining this E15.5–E18.5 time point allowed analysis of primary effector CD8 T cells in the spleen at the height of fetal antigen exposure. OT-1 T cell activation was also examined in OVA-grafted animals at a similar time point (∼15–20 d after grafting) as a control.

Donor OT-1 cells extensively divided in the spleens in all cases, with a trend toward reduced proliferation of OT-1 cells in pOVA^OT-1^ mice compared with gOVA^OT-1^ mice (Fig. 10 B). Although this effect reached statistical significance only for pOVA^OT-1^ mice receiving 3 × 10^5^ cells in this experiment, this trend was consistent with our prior observations in parous mice (Fig. 1 B), suggesting overall that the signals promoting cell division may be different between the settings of pregnancy and skin transplantation. Nevertheless, expression of TOX, PD-1 (Fig. 10 C), and Aiolos (Fig. 10 D) was higher in divided primary effector OT-1 T cells recovered from pregnant mice than in those from skin-grafted animals, although differences in SATB1 expression between pOVA^OT-1^ and gOVA^OT-1^ mice were not yet observed (Fig. 10 E). Altogether, these data collectively suggest that exhaustion programming begins during effector differentiation.

Finally, to examine induction of function, we examined cytokine production by intracellular cytokine staining. Although OT-1 T cells primed by skin grafts were capable of producing IFN-γ and/or TNF, function of OT-1 T cells from pOVA mice was poor compared with OT-1 cells in gOVA mice (Fig. 10 F). Poor cytokine production in effector OT-1 T cells recovered from pregnant mice was not dramatically altered by precursor frequency, as similar results were obtained from mice that received adoptive transfer of either 3 × 10^6^ or 3 × 10^5^ OT-1 T cells before mating (Fig. 10 F). Altogether, these results suggest that constraints on the functionality of maternal CD8 T cells are established early after antigen encounter during pregnancy.

## Discussion

We aimed to improve our understanding of the molecular mechanisms that govern CD8 T cell fate and function after exposure to fetal antigen during pregnancy. Although much of our current knowledge of CD8 T cell differentiation derives from studies of T_MEM_ cells that evolve following acute infection, antigen encounter in tumors or during chronic viral infections can generate T_EX_ cells with substantially attenuated function (Wherry et al., 2007; McLane et al., 2019; Schietinger et al., 2016; Scott et al., 2019). In the current studies, we asked whether similar molecular mechanisms underpin maternal T cell tolerance of the fetus in pregnancy. Study of parous mice demonstrated that maternal fetal-specific CD8 T cells differentiate into antigen-experienced T cells that possess limited functionality compared with bona fide T_MEM_ cells. Notably, maternal CD8 T cells possessed phenotypic and transcriptional features of T_EX_ cells. Moreover, we identified both NFAT-dependent and likely NFAT-independent circuits that contribute to induction of CD8 T cell exhaustion during pregnancy. Although many features of exhaustion were shared between Preg-T_EX_ and T_EX_ cells generated in other settings, CD8 T cell exhaustion in pregnancy persisted after antigen clearance and was associated with T cell hypofunction despite more modest expression of NFAT-dependent circuitry such as TOX and PD-1. Thus, these data identify pregnancy as a previously unappreciated setting where T cell exhaustion may occur.

One set of observations here identified the NFAT/TOX/PD-1 transcriptional circuit as an exhaustion-driving mechanism in pregnancy, linking this pathway to T cell exhaustion arising in cancer and chronic infection (Scott et al., 2019; Khan et al., 2019; Alfei et al., 2019; Seo et al., 2019). Consistent with these prior studies, we found that TOX and PD-1 were induced downstream of NFAT during pregnancy as maternal T cells were exposed to antigen and underwent effector differentiation. However, compared with another setting of robust induction of exhaustion, LCMV clone 13 infection, induction of TOX and PD-1 expression was reduced in Preg-T_EX_ cells. These differences in PD-1 and TOX expression could reflect differences in antigen dose or duration between pregnancy and chronic viral infection or potential inflammatory differences. What drives this moderated PD-1 and TOX in pregnancy compared with chronic viral infection is unclear. It is possible that differences in NFAT signals due to altered patterns of antigen exposure in pregnancy could account for this less robust induction of these exhaustion-associated pathways, but other possibilities, including inflammation, antigen-presenting cell differences, or altered costimulation, could also contribute. Moreover, a role for fetal microchimerism or persistent antigen presentation after pregnancy (McCloskey et al., 2011) cannot be excluded. Nevertheless, these findings suggest that the NFAT/TOX/PD-1 transcriptional circuit is a key feature of exhaustion induction in Preg-T_EX_ cells linking one part of the exhaustion induction program to other settings of T cell exhaustion.

Whereas differences in initial exhaustion programming may exist between pregnancy and chronic infection, differences in maintenance of the exhaustion program may also contribute to variation in exhaustion severity. For example, it is possible that Preg-T_EX_ cells have reduced expression of elements of the exhaustion program because these cells are “recovering” after antigen withdrawal. Indeed, so-called recovered T_EX_ (REC-T_EX_) cells have recently been shown to differentiate from T_EX_ cells in the LCMV clone 13 model when T_EX_ cells were removed from chronic antigen stimulation (Abdel-Hakeem et al., 2021). Notably, REC-T_EX_ cells had reduced expression of many phenotypic and transcriptional elements of the T_EX_ cell program, including TOX and PD-1, and these cells acquired elements of T_MEM_ cells. However, recall responses and the overall functionality of REC-T_EX_ cells remained substantially inferior to those of T_MEM_ cells due to persistence of T_EX_ cell epigenetic identity. Pregnancy may thus more closely mirror this situation where T cell exhaustion is established and then altered once antigen is cleared. Future longitudinal studies that map the epigenome of Preg-T_EX_ cells throughout pregnancy and the postpartum period to test this idea should be informative.

Despite the induction of TOX and PD-1 during pregnancy, our data collectively suggest that the NFAT/TOX/PD-1 exhaustion circuit in Preg-T_EX_ cells is not strictly coupled to maternal T cell function. Indeed, Preg-T_EX_ cells remained hypofunctional despite reductions of TOX and PD-1 expression in the setting of FK506 treatment. Overall, these results are consistent with experiments in FK506-treated tumor-bearing mice (Scott et al., 2019) and also recapitulate the modest improvements in T_EX_ effector function observed by Khan et al. (2019) in TOX knockout P14 cells. Despite the consistency of these observations, the role that TOX and PD-1 play in programming T_EX_ effector function—explicitly cytokine production—remains incompletely understood. Indeed, our studies identify only a weak inverse correlation between IR expression and expression of TNF. Together with data from FK506 treatment, these observations suggest that IRs serve to tune T cell functionality around a set point that is established by other mechanisms. At present, the molecular determinants of this functionality set point in Preg-T_EX_ cells are unknown, and our data suggest that NFAT-independent transcriptional programs may contribute. It will be interesting in the future to better define how NFAT-dependent and -independent mechanisms contribute Preg-T_EX_ cell differentiation and function.

In addition to up-regulation of the NFAT/TOX/PD-1 pathways, translational repression was another major conserved feature of exhaustion biology identified here. This “node” of exhaustion biology was identified in early transcriptional analyses of T_EX_ cells (Wherry et al., 2007), but it has remained poorly understood. In the setting of Preg-T_EX_ cells, this effect was manifested by down-regulation of ∼30 coregulated genes encoding ribosomal proteins. Although there was some variation in the specific ribosomal genes altered when comparing cancer, chronic viral infection, and pregnancy, these differences may reflect the relatively low expression of many of these genes and/or the context-specific solutions T_EX_ cells find to the need to limit protein translation. The need to restrain protein translation may reflect the bioenergetic deficiencies of T_EX_ cells (Bengsch et al., 2016; Klein Geltink et al., 2018; Scharping et al., 2021), necessitating limitation of the most energy-dependent cellular process. Although it is difficult to draw conclusions about individual ribosomal or protein translation genes, the down-regulation of this coregulated module in different exhaustion contexts likely represents a conserved biological mechanism. Although multiple alterations in the control of protein translation appear to be a common feature of T_EX_ cells in multiple contexts (Wherry et al., 2007; Bengsch et al., 2018; Schietinger et al., 2016), the connection to an NFAT-mediated down-regulation of ribosome biogenesis may represent a regulatory axis that could be amenable to manipulation in the future. The extent to which non-NFAT signals influence ribosomal biogenesis remains unclear. Whether repression of ribosomal gene expression in T_EX_ cells can be reversed may also have implications for recovering robust and sustained responses from such cells.

An additional goal of this study was to evaluate the clinical consequences of pregnancy-induced T cell hypofunction. In μMT mice where antibody was not a factor, skin graft survival was better in mice alloimmunized by pregnancy than in mice sensitized by prior skin transplant. Our results largely recapitulate those of Suah et al. (2021), who similarly attributed prolonged survival of an F1 heart transplant in allogeneically mated parous mice to maternal T cell hypofunction induced by pregnancy. Similar to our studies, graft survival in mice receiving a heart transplant was most marked in μMT mice where pregnancy-induced antibody could not develop or participate in graft rejection. However, despite heart transplant acceptance by parous μMT mice in these studies, skin grafts were rejected in parous μMT mice with kinetics identical to that in naive mice. The authors attribute these differences between rejection of heart versus skin transplants to the increased susceptibility of skin grafts over heart grafts to rejection. Skin transplant results from this previous work in μMT mice is largely consistent with the current studies, where the tempo of skin graft rejection in antigen-experienced mice is similar to that of naive mice. Because Suah et al. did not report on skin graft outcomes in parous μMT mice compared with previously skin grafted animals, it is unclear whether this skin graft rejection is accelerated or delayed compared with a T_MEM_ cell response in this previous work. Nevertheless, our studies collectively demonstrate that key changes in maternal T cells during pregnancy may alter future allograft rejection. Of note, Suah et al. also showed that accelerated heart graft rejection was restored in parous sIgKO mice that cannot secrete antibodies, suggesting a role for B cells in the restoration of function in maternal T cells in some settings of allograft rejection. It will be important to determine how B cells specifically influence the functionality of T cells in parous mice in future studies, as this work may pave the way for novel immunomodulation strategies for parous transplant recipients.

We also wanted to understand how these studies in mice might extend to humans. We therefore compared graft outcomes in human transplant recipients who had been alloimmunized by either transplant or pregnancy. Consistent with the results of mouse models, parous transplant recipients have improved graft outcomes compared with other alloimmunized populations. Although we attribute improved graft outcomes in parous mice and humans to the presence of T_EX_ cells, other cell populations, such as expanded populations of regulatory T cells, may also contribute. Differences in kidney transplant survival in the United Network for Organ Sharing (UNOS) cohort may also be due to other confounding variables. For example, we could not control for the potential contribution of mismatched minor antigens (Gratwohl et al., 2008; Heinold et al., 2008) or overall immunosuppression burden (in the nonoffspring patient cohort) on graft survival. The analyzed cohorts were also too small to match for donor type or other potential confounders such as the sex of the child or the kidney donor. Nevertheless, our observation of prolonged graft survival in both human and mouse transplant models aligns with our other experimental data demonstrating maternal CD8 T cell hypofunction as well as with the work of other investigators (Suah et al., 2021). The identification of Preg-T_EX_ cells in parous women will be an important next step in determining the clinical relevance of this population.

In summary, these studies demonstrate that pregnancy induces the differentiation of exhausted maternal CD8 T cells that persist and impact postpartum immune responses. Although Preg-T_EX_ cells share a core program of T cell exhaustion with other contexts, our studies also identify distinct differences in the transcriptional program that are likely driven by context-dependent signals. Pregnancy thus presents a unique physiological context in which to study the biology of hypofunctional T cells, particularly in the setting of antigen clearance. In conclusion, immunological mechanisms of maternal–fetal tolerance are retained after pregnancy is complete, with significant implications for the long-term health of women.

## Materials and methods

### Animals

C57BL/6J (CD45.2, H-2^b^), B6.SJL-*Ptprc^a^ Pepc^b^*/BoyJ (“CD45.1,” H-2^b^), C57BL/6-Tg(TcraTcrb)1100Mjb/J (“OT-1,” CD45.2, H-2^b^), C57BL/6-Tg(CAG-OVA)916Jen/J (“Act-mOVA,” H-2^b^), and B6.129S2-*Ighm^tm1Cgn^*/J (“µMT,” H-2^b^) were all purchased from The Jackson Laboratory and housed in a pathogen-free facility at the University of Pennsylvania or at the University of Alabama at Birmingham. All mice in these experiments were healthy adults (8–12 wk old), had undergone no prior experimentation, and were randomly allocated to experimental groups. Female mice were group housed (maximum of five females/cage). Virgin females were housed individually overnight with male mice, visually inspected for copulation plugs the following morning (E0.5), and returned to female-only holding cages, separate from male partners for the remainder of gestation. The specific genotype of mating pairs for each experiment is noted in the Results. Pregnant females were inspected daily between E18.5 and E21.5 to note the date of parturition and enumeration of live pups. P14 TCR transgenic mice were bred at the University of Pennsylvania and cells were injected into C57BL/6 mice purchased from the National Cancer Institute. All animal care was provided according to protocols reviewed and approved by the institutional animal care and use committees at the University of Pennsylvania and the University of Alabama at Birmingham.

### Human national registry data

National registry data from the OPTN were used for analysis of human kidney transplant recipients. The OPTN includes data, submitted by its members, on all donor, wait-listed candidates, and transplant recipients in the United States. The Health Resources and Services Administration of the U.S. Department of Health and Human Services provides oversight of the activities of the OPTN contractor. Data from the transplant registry were used according to a data use agreement signed with the OPTN. Given the de-identified nature of the registry data, UNOS data are exempt from review by the institutional review board.

### Skin transplantation

Recipient mice were anesthetized with i.p. injection of ketamine and xylazine, and the chest wall and flank were shaved. After antiseptic skin preparation, a 1 × 1–cm area of skin was removed from the s.c. tissues. Donor skin grafts were prepared from euthanized mice and subsequently sutured into the prepared recipient bed using interrupted 5-0 Maxon suture (Covidien). Bandages were secured in place and removed after 7 d for observational graft survival experiments. Skin grafts were inspected daily from bandage removal until termination of the experiment. Skin graft rejection was recorded to occur on the day that >75% of the graft was necrotic.

### LCMV infection

The LCMV clone 13 strain was propagated in baby hamster kidney cells, and titers were determined using plaque assays on Vero cells as previously described (Odorizzi et al., 2015). Mice were infected i.v. with 4 × 10^6^ PFU of LCMV clone 13 one day after adoptive transfer of 10^3^ naive transgenic P14 cells. Spleens were harvested at indicated time points for flow cytometry analysis.

### Adoptive transfer and proliferation assays

Single-cell suspensions at the designated concentration were prepared from OT-1, CD45.2 splenocytes; suspended in 200 µl of PBS; and injected into anesthetized CD45.1 recipient animals via a retro-orbital route. For proliferation assays, cells were labeled with 10 µM CTV (Invitrogen) before transfer.

### Flow cytometry and cell sorting

Cell staining was performed by incubating single-cell suspensions with the following fluorophore-conjugated antibodies purchased from BD Biosciences (PE-CF594 anti-CD127 [SB/199], BV711 anti-Lag3 [C9B7W], PE-CF594 anti-Aiolos [S48-791], Alexa Fluor 647 anti-SATB1 [14/SATB1], PE anti–phospho-ERK1/2 [MILANBR]); eBioscience (A700 anti-CD62L [MEL-14], PE–cyanine 5.5 [Cy5.5] anti-CD4 [RM4-5], APCeF780 anti-CD8 [53-6.7], PE-eFluor 610 anti-Eomes [Dan11mag], PE anti-TOX [TXRX10]); and BioLegend (BV605 anti-CD4 [RM445], BV650 anti-CD45.2 [104], BV711 anti–IFN-γ [XMG1.2], BV785 anti-CD44 [IM7], PE/Cy7 anti-CD279 [PD-1; RMP1-30], A700 anti-CD45.1 [A20], FITC anti–TNF-α [MP6-XT22], PE-Cy7 anti-CD38 [90]). Intracellular cytokine staining was performed after fixation and permeabilization using an eBioscience Foxp3/Transcription Factor Staining Buffer Set kit per the manufacturer’s instructions after in vitro stimulation with peptide. Data were collected for live cells according to viability labeling with LIVE/DEAD Fixable Aqua Dead Cell staining (Thermo Fisher Scientific). All data were collected on an LSRII cytometer (BD Biosciences) and analyzed with FlowJo software (version X.0.7; BD Biosciences). For experiments that involved T cell sorting, CD8^+^ cells were enriched before antibody staining using the EasySep Mouse CD8+ T Cell Isolation kit (catalog no. 19853; STEMCELL Technologies) according to the manufacturer’s instructions.

### In vitro peptide stimulation

Freshly isolated splenocytes from postpartum mice were suspended in RPMI at 5 × 10^6^/ml and co-cultured for 5 h with SIINFEKL peptide at 0.4 µg/ml (GenScript) before cell surface staining and flow cytometric analysis (protocol above).

### In vivo cytotoxicity

4 × 10^6^ target splenocytes from Act-mOVA and B6 mice were labeled with 3.33 or 0.25 µM of CTV, respectively, and injected into virgin control or OVA skin-grafted mice 7 d after transplantation. Target cells were enumerated in the spleen of all recipient mice 24 h after transfer using flow cytometry.

### FK506 injection

FK506 (Astellas Pharma Inc.) was injected daily (1 mg/kg i.p.) into pregnant mice.

### Phospho-flow analysis

Single-cell suspensions of murine splenocytes were stained with Aqua Fluorescent Reactive Dye (1:500, catalog no. L34966; Invitrogen), washed twice with PBS, and resuspended in culture RPMI. Cell number was adjusted to 5 × 10^6^ cells/ml in medium and plated to 24-well plates. Cells were stimulated with PMA (40 nM) for 15 min at 37°C or with SIINFEKL peptide (0.4 μg/ml) for 5 min at 37°C. Cells were then fixed by addition of IC Fixation Buffer (Invitrogen) at room temperature in the dark for 1 h and permeabilized in 1 ml of ice-cold methanol for 30 min at 4°C. Cells were washed, then stained for 1 h at room temperature with anti-CD4, anti-CD45.2, anti-pErk1/2, anti-CD8, and anti-CD45.1 antibodies. Cells were washed twice with staining buffer and analyzed using the BD LSR II flow cytometer (BD Biosciences).

### Serum transfer

Serum donor mice included naive, pOVA, and gOVA animals. Age-matched 8–12-wk-old C57BL/6J female mice (B6 mice; The Jackson Laboratory) were used to produce serum. To make pOVA antibody (pOVA Ab), B6 mice were mated with Act-mOVA male mice to generate pregnant mice (pOVA; *n* = 5), and blood was collected from mice 30 d after delivery. Serum was prepared, pooled, and stored at −80°C. To make gOVA antibody (gOVA Ab), B6 mice (*n* = 5) were transplanted with an OVA skin graft, and serum was prepared as above 30 d after transplant. Serum of naive B6 mice (*n* = 5) was prepared as a control. The concentration of anti-OVA was measured by ELISA. B6.129S2-*Ighm^tm1Cgn^*/J mice (μMT mice; The Jackson Laboratory) were mated with Act-mOVA male mice to generate pOVA μMT mice. Serum containing ∼5,000 U/ml of anti-OVA antibody was injected i.p. into pOVA μMT mice.

### ELISA

Serum samples were prepared from mice and diluted 100-fold in PBS (sample diluent buffer) before incubation on capture plates. Positive control sera were prepared from naive virgin C57BL/6J and µMT mice that were immunized on day 2 and day 7 with 1 µg chicken OVA (Invitrogen) in CFA (1:1 vol/vol) via i.p. injection. Negative control sera were prepared similarly from naive virgin C57BL/6J and µMT mice that received PBS injection with CFA on day 2 and day 7. Sera from negative and positive control mice were collected 30–45 d after injection. Sera from gOVA and pOVA mice were similarly prepared 50–60 d after grafting or timed mating, respectively.

A semiquantitative anti-OVA ELISA was used for Fig. S1 A. 96-well plates were coated with 50 µl of 10 µg/µl OVA antigen (EndoFit; InvivoGen) and incubated overnight at 4°C. Plates were washed with PBS the following morning after 2-h incubation at room temperature. Plates were then blocked, incubated overnight with the blocking agent, and washed the following day. 50 µl of serum (1:4 dilution) was added to wells and incubated at room temperature for 2 h. Plates were washed, and 50 µl of Ig-HRP (1:4,000 dilution) was added to individual wells. Plates were incubated for another 2 h at room temperature and washed. Plates were then incubated for 20 min at room temperature after addition of 100 µl of tetramethylbenzidine. The reaction was then stopped, and plates were read on a VMax kinetic microplate reader (Molecular Devices).

A quantitative anti-OVA ELISA kit that measured total IgA, IgM, and IgG was purchased from Alpha Diagnostic International (catalog no. 600-100-OGG) and used for experiments in Fig. S1 B. ELISA was performed according to the manufacturer’s instructions. Serum concentration of anti-OVA antibody in samples was calculated after performing linear regression on the standard curve created from the standard reagents included in the ELISA kit.

### Quantitative real-time RT-PCR (qRT-PCR)

For validation of RNA-seq results, total RNA of sorted CD8^+^ cells from naive, gOVA, and pOVA animals was isolated using an RNeasy Plus Micro Kit (Qiagen). cDNA was made from total RNA using the SMART-Seqv4 Ultra Low Input RNA Kit for Sequencing (Takara Bio). mRNA expression for genes encoding *Tox* and *Gapdh* were measured using qRT-PCR. qRT-PCR was performed in triplicate with TaqMan Fast Advanced Master Mix (Life Technologies) following the recommended protocols in the QuantStudio 6 Real-Time PCR System (Applied Biosystems). qRT-PCR was performed using ready-to-use primer and probe sets predeveloped by Applied Biosystems. TaqMan Gene Expression Assays were used to quantify *Tox* (Mm0045523_ml) and *Gapdh* (Mm99999915_g1). For RT-PCR assays measuring ribonucleic proteins, RNA and cDNA were prepared from sorted CD8^+^ cells from naive, gOVA, and pOVA animals using a Cells-to-cDNA kit (Thermo Fisher Scientific). Results were normalized to either *Gapdh* (or *Actin*) levels using the formula cycle threshold (ΔCt) = Ct of target gene − Ct of *Gapdh*. The mRNA level of the control group was used as the baseline; therefore, the comparative ΔCt (ΔΔCt) was calculated using the formula ΔΔCt = ΔCt of target gene − ΔCt of the baseline. The fold change of mRNA level was calculated as fold = 2^−ΔΔCt^.

### Bulk RNA-seq

CD8^+^CD45.2^+^ cells from OT-1 mice were injected into CD45.1 recipients as described. 50–60 d after injection, CD8^+^CD45.2^+^ cells were sorted from naive^OT-1^, OVA-grafted^OT-1^ (gOVA^OT-1^), and OVA-parous^OT-1^ (pOVA^OT-1^) mice (10 mice in each group). Cells were washed with PBS once, frozen with lysis buffer, and stored at −80°C. Cells from three or four mice within the same group were combined into one sample to make a final three samples per group before RNA extraction. RNA was extracted using the RNeasy Plus Micro Kit (Qiagen), and cDNA was synthesized using the SMART-Seq V4 Ultra Low Input RNA Kit for Sequencing (Takara Bio). A cDNA library was constructed using the Nextera XT DNA Library Prep Kit (Illumina) and quantified by quantitative PCR using a KAPA Library Quantification Kit (Kapa Biosystems). The products were sequenced on an Illumina NextSeq 500 platform (Illumina) using a NextSeq 500/550 v2 kit, following the manufacturer’s instructions. RNA-seq data are available via the Gene Expression Omnibus (accession no. GSE188808).

### Differential gene expression analysis

Transcripts were aligned to the mouse reference genome (*Mus musculus*; University of California, Santa Cruz Genome Browser mm10) using the STAR aligner (STAR_2.5.0b), and Cufflinks was used to assemble transcripts and quantify transcript abundance. Differential gene expression analysis of assembled transcriptomes for each of the nine samples (three samples per group) was subsequently performed using EdgeR.

### GSEA

GSEA software (4.0.3) was downloaded from the Broad Institute (https://www.gsea-msigdb.org/gsea/index.jsp), and preranked GSEA was performed on selected gene sets and ranked gene lists. Gene set files were downloaded from the Molecular Signatures Database or prepared manually as gene matrix expression files (.GMX) from either published or unpublished data. Ranked gene lists were generated from published and unpublished datasets by ordering genes based on the rank metric: signed fold change × −log_10_ P value.

### Biological pathway and network analyses

The Metascape gene annotation and analysis resource (http://metascape.org; Zhou et al., 2019) was used to identify enriched biological processes from Gene Ontology, KEGG, and Reactome datasets for selected gene sets. Additional pathway analyses and network construction were performed using the EnrichmentMap application of Cytoscape (3.7.2; Reimand et al., 2019; Merico et al., 2010) as well as gene sets of biological processes compiled by the Bader laboratory. Predicted regulons of the coregulated ribosomal proteins identified by Metascape in the Ribosome pathway (KEGG mmu03010) were subsequently identified and mapped using the iRegulon application of Cytoscape (3.7.2; Janky et al., 2014).

### Propensity score–matched models of registry data

Analyses of human kidney transplant recipients were performed using national registry data from the OPTN. A cohort was generated to mirror the experimental model of the primary mouse study (Fig. 1), including all female recipients of offspring donor kidneys as well as all recipients of a second haplotype-matched kidney transplant (wherein the first and second donors were required to be HLA identical). Recipients were excluded if they had a maximum panel reactive antibody >0%, if they were ABO blood type incompatible with any transplanted donor kidney, or if they were <35 yr old (i.e., the youngest age of a female recipient of an offspring kidney) at the time of transplant.

### Statistical analysis

#### Murine experiments

Continuous variables were compared using the Student’s *t* test statistic (unpaired, two-tailed) in Microsoft Excel 2016. An assumption of unequal variance was included in the *t* test calculation. Graft survival was compared between groups using the method of Kaplan and Meier and analyzed using the log-rank test statistic in SPSS Statistics version 24. P values <0.05 were deemed statistically significant. The figures and figure legends provide precise sample sizes and statistical results for each experiment.

#### Human national registry data

Statistical analyses were performed using STATA version 15.0 (StataCorp) with two-sided hypothesis testing and a P value <0.05 as the criteria for statistical significance. We performed propensity score matching to balance important recipient characteristics across each exposure group, using logistic regression to generate a propensity score on recipient age, race, and diabetes status. We implemented 1:1 nearest neighbor matching without replacement, using a caliper of 0.01 with common support. We assessed for balance between the matched groups using the standardized difference (<10% was accepted), Rubin’s B (<25% was accepted), and Rubin’s R (0.2–5 was accepted). Cox proportional hazards regression was used to estimate the hazard of all-cause allograft failure (a composite outcome of allograft failure and mortality) across the matched groups. Robust sandwich estimation of the SEs was used to account for clustering within the matched pairs. The proportional hazards assumption was assessed with statistical testing based on the Schoenfeld residuals.

### Online supplemental material

Fig. S1 quantifies anti-OVA antibody generated through immunization by pregnancy or skin graft transplantation. Fig. S2 enumerates the selection of human kidney transplant recipients from the UNOS dataset for propensity score matching and graft survival analysis. Table S1 shows the HLA allele matching for transplant recipients included in the graft survival analysis. Table S2 shows baseline demographic and clinical characteristics of human kidney transplant recipients included in the graft survival analysis. Table S3 lists differentially expressed genes between OVA-parous and OVA-grafted mice.

## Supplementary Material

Table S1 lists HLA specificities of donors and recipients.

Table S2 lists baseline characteristics of human offspring recipients compared with recipients of a second haplotype-matched kidney (where the first and second donors are HLA identical) using national registry data.

Table S3 shows differentially expressed genes pOVA versus gOVA.
