# Supplementary material for: NFAT-dependent and -independent exhaustion circuits program maternal CD8 T cell hypofunction in pregnancy
Source: J Exp Med. 2021 Dec 9;219(1):e20201599. doi: 10.1084/jem.20201599 (PMC8666877; doi:10.1084/jem.20201599)
Supplement: Table S2 — lists baseline characteristics of human offspring recipients compared with recipients of a second haplotype-matched kidney (where the first and second donors are HLA identical) using national registry data. [file JEM_20201599_TableS2.docx]

Table S2. Baseline characteristics of human offspring recipients compared with recipients of a second haplotype-matched kidney (where the first and second donors are HLA identical) using national registry data

|  | Offspring recipients (*n* = 3,001) | Nonoffspring recipients (*n* = 31) | P value |
| --- | --- | --- | --- |
| **Recipient characteristics** | | | |
| Median age, yr (IQR) | 59 (53–65) | 49 (44–57) | <0.001 |
| African American race, *n* (%) | 746 (25%) | 1 (3%) | 0.005 |
| History of diabetes, *n* (%) | 1,106 (38%) | 6 (19%) | 0.032 |
| Median dialysis vintage, d (IQR) | 319 (0–684) | 405 (0–805) | 0.878 |
| Median body mass index, kg/m^2^ (IQR) | 28 (24–32) | 26 (23–29) | 0.133 |
| Cause of end-stage renal disease, *n* (%) | | | <0.001 |
| Diabetes | 890 (30%) | 1 (3%) |  |
| Hypertension | 781 (26%) | 0 (0%) |  |
| Glomerular disease | 498 (17%) | 5 (16%) |  |
| Cystic disease | 223 (7%) | 1 (3%) |  |
| Other | 389 (13%) | 24 (77%) |  |
| **Donor characteristics** | | | |
| Median age, yr (IQR) | 33 (28–39) | 38 (23–48) | 0.103 |
| African American race, *n* (%) | 752 (25%) | 0 (0%) | 0.001 |
| Median body mass index, kg/m^2^ (IQR) | 27 (24–30) | 25 (22–28) | 0.006 |
| **Immunological characteristics** | | | |
| ABO blood type identical, *n* (%) | 2,432 (81%) | 23 (74%) | 0.334 |
| Induction immunosuppression, *n* (%) | | | 0.640 |
| None | 994 (33%) | 9 (29%) |  |
| Depleting | 1,202 (40%) | 15 (48%) |  |
| Nondepleting | 805 (27%) | 7 (23%) |  |
| Calcineurin inhibitor immunosuppression, *n* (%) | | | 0.306 |
| Tacrolimus | 2,133 (71%) | 19 (61%) |  |
| Cyclosporine | 682 (23%) | 8 (26%) |  |
| Both | 18 (1%) | 0 (0%) |  |
| Neither | 168 (5%) | 4 (13%) |  |
